# Signaling pathways in liver cancer: pathogenesis and targeted therapy

**DOI:** 10.1186/s43556-024-00184-0

**Published:** 2024-05-31

**Authors:** Yangtao Xue, Yeling Ruan, Yali Wang, Peng Xiao, Junjie Xu

**Affiliations:** 1 Key Laboratory of Laparoscopic Technology of Zhejiang Province, Department of General Surgery, Sir Run-Run Shaw Hospital, Zhejiang University School of Medicine, Hangzhou, 310016 China; 2 Sir Run-Run Shaw Hospital, Zhejiang University School of Medicine, Hangzhou, 310016 China; 3 National Engineering Research Center of Innovation and Application of Minimally Invasive Instruments, Hangzhou, 310016 China; 4 Zhejiang Minimal Invasive Diagnosis and Treatment Technology Research Center of Severe Hepatobiliary Disease, Zhejiang Research and Development Engineering Laboratory of Minimally Invasive Technology and Equipment, Hangzhou, 310016 China; 5 Zhejiang University Cancer Center, Hangzhou, 310058 China; 6 Liangzhu Laboratory, Zhejiang University Medical Center, Hangzhou, 311121 China

**Keywords:** Liver cancer, Hepatocellular carcinoma (HCC), Targeted therapy, Signaling pathways, Sorafenib

## Abstract

Liver cancer remains one of the most prevalent malignancies worldwide with high incidence and mortality rates. Due to its subtle onset, liver cancer is commonly diagnosed at a late stage when surgical interventions are no longer feasible. This situation highlights the critical role of systemic treatments, including targeted therapies, in bettering patient outcomes. Despite numerous studies on the mechanisms underlying liver cancer, tyrosine kinase inhibitors (TKIs) are the only widely used clinical inhibitors, represented by sorafenib, whose clinical application is greatly limited by the phenomenon of drug resistance. Here we show an in-depth discussion of the signaling pathways frequently implicated in liver cancer pathogenesis and the inhibitors targeting these pathways under investigation or already in use in the management of advanced liver cancer. We elucidate the oncogenic roles of these pathways in liver cancer especially hepatocellular carcinoma (HCC), as well as the current state of research on inhibitors respectively. Given that TKIs represent the sole class of targeted therapeutics for liver cancer employed in clinical practice, we have particularly focused on TKIs and the mechanisms of the commonly encountered phenomena of its resistance during HCC treatment. This necessitates the imperative development of innovative targeted strategies and the urgency of overcoming the existing limitations. This review endeavors to shed light on the utilization of targeted therapy in advanced liver cancer, with a vision to improve the unsatisfactory prognostic outlook for those patients.

## Introduction

Annually, liver cancer emerges as a highly prevalent form of malignancy, resulting in a considerable amount of fatalities. In global cancer statistics for 2020, liver cancer stands as the sixth most frequently diagnosed type of cancer. Hepatocellular carcinoma (HCC) accounts for almost 90% of the approximately 906,000 liver cancer cases diagnosed globally [1, 2]. Cholangiocarcinoma (CCA) is the second most prevalent primary liver malignancy after HCC and is categorized into three subtypes: distal CCA (dCCA), perihilar CCA (pCCA), and intrahepatic CCA (iCCA) [3]. A recent study conducted in the US reported an increasing trend in the incidence of CCA with age [4]. Many ongoing studies have focused primarily on HCC because of its high incidence among liver cancers. HCC ranks third in cancer-related mortality, with a merely 18% of 5-year survival rate [5].

Unlike other solid tumor varieties, such as those of the breast, lung, and colon, HCC arises from chronically damaged tissues resulting from a combination of factors [6]. These factors include both internal and external elements, such as genetic predisposition, viral or non-viral factors, and cellular microenvironment [1]. For instance, alterations in genes that drive cancer can activate several signaling pathways downstream, leading to increased cell growth and a reduction in programmed cell death [7]. Furthermore, additional risk elements, like infection with HBV or HCV and fatty liver, which result in chronic inflammation, may contribute to HCC development as well [1]. From an overarching perspective, the tumor microenvironment (TME) of HCC—comprising cancer cells, immune cells, cytokines, and the extracellular matrix—is crucial in facilitating signaling pathways that promote tumors and aid in developing resistance to treatment [8, 9].

Although surgical options, such as liver transplantation or resection, ablation, transcatheter arterial chemoembolization (TACE), and even radiotherapy, are available for patients with HCC [10], numerous patients are diagnosed at an intermediate or advanced stage and require systemic therapy, including targeted therapy [11]. Therefore, investigating the molecular basis of the pathogenesis of liver cancer, especially HCC, and identifying potential therapeutic targets is crucial for developing interventions or effective treatment strategies, enhancing the prognosis of patients.

Given the complexity of HCC pathogenesis, targeted therapy remains immature and the application is highly limited despite numerous studies underway. This review aims to collate information on pro-tumor pathways of liver cancer and the progression of pathway-specific inhibitors, thereby offering insights for the future development of targeted therapies. Hence, we adopt a broad perspective, striving to provide a comprehensive exposition of the pathways involved in the progression of liver cancer and the correlated targeted treatments especially tyrosine kinase inhibitors (TKIs), due to their widespread use in the therapeutic management of HCC. Furthermore, we additionally elaborate on the mechanisms using sorafenib as an illustrative example.

In this article, we first categorize signaling pathways implicated in liver cancer pathogenesis into two groups, based on whether it is activated by growth factors together with their receptors and summarize the current findings of each one. Then we organize and describe their related molecular inhibitors, including their current research and the clinical study if they have participated in. Considering the significant status of TKIs relative to other inhibitors, we subsequently dedicate two sections to discuss this category of medications and the common mechanisms underlying resistance encountered during their clinical use. Overall, this review highlights potential avenues for future advancements in the targeted therapy of liver cancer.

## Growth factor receptor-related signaling pathways in liver cancer

**Fig. 1:**
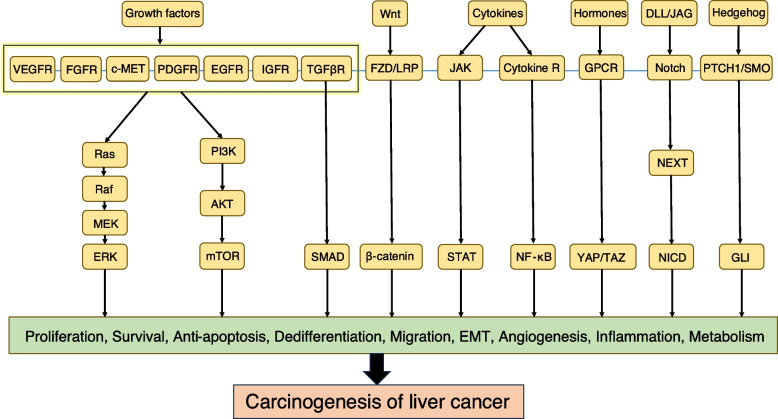
An overview of signaling pathways in the pathogenesis of liver cancer. Here we present a diagram of the pathways and their key components related to the occurrence of liver cancer, including growth factor receptor-related signaling pathway, Wnt-β-catenin, JAK/STAT, Hedgehog, Hippo, Notch and NF-κB signaling pathway

**Fig. 2:**
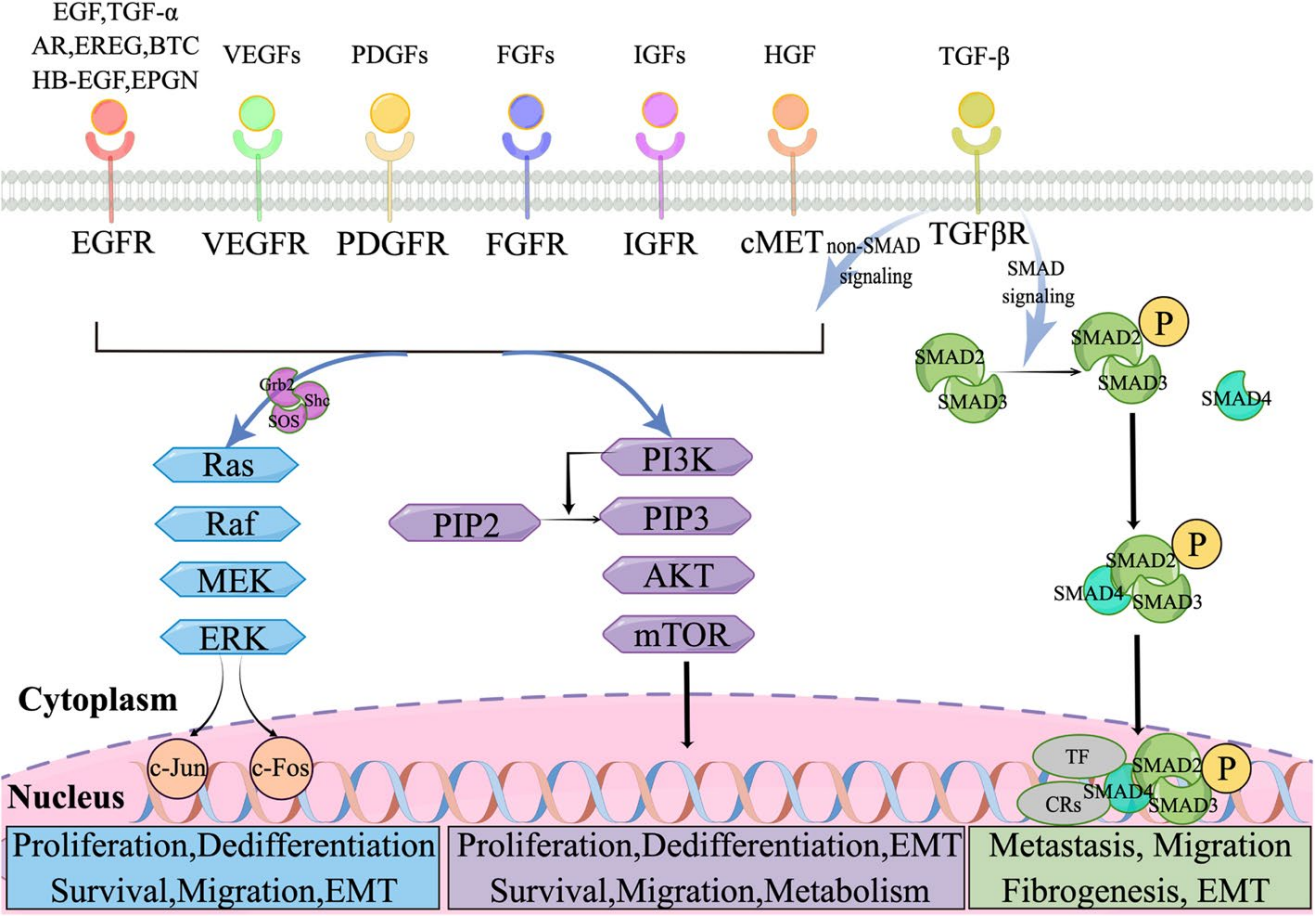
Growth factor receptor-related signaling pathways. Various growth factors act as external signals that trigger the activation of tyrosine kinase receptors such as VEGFR, FGFR, c-MET, PDGFR, EGFR, IGFR, and TGFβR. Excluding the unique case of TGFβR, the transmission of signals to the cell nucleus mainly involves two pathways: the Ras/Raf/MEK/ERK and the PI3K/AKT/mTOR cascades, which ultimately influence the transcription of specific genes. TGF-β signaling can be categorized into SMAD pathway and non-SMAD pathway. The latter falls under the previously mentioned pathways. In the case of SMAD-dependent signaling, TGFβR phosphorylates SMAD2/3, which then associates with SMAD4. This complex moves to the nucleus to modulate gene transcription. By figdraw

In fact, a variety of carcinogenic pathways are implicated in liver cancer pathogenesis. To circumvent the monotony that might arise from listing each pathway one by one, we categorize these pathways into two groups (Fig. 1), based on whether they are activated by the upstream growth factor-related receptors. Our discussion initially centers on pathways related to growth factor receptors (Fig. 2). In this segment, we find that the activation of growth factor receptors not only stimulate downstream pathways such as the most commonly seen Ras/Raf/MEK/ERK signaling [12] and PI3K/AKT/mTOR signaling [13] directly, but also promote tumorigenesis by alternative mechanisms. Consequently, we sequentially elaborate on the regulatory role of these growth factor receptors and their corresponding binding ligands in the onset of liver cancer and elucidate how their downstream pathways further contribute to carcinogenesis.

### Activation of tyrosine kinase receptors and specific ligands

#### VEGF

Angiogenesis refers to the process where new blood vessels emerge within tumors, particularly indicating the branching out of new vessels from those already established [14]. Angiogenesis is widely recognized as a hallmark of tumor development [15]. Vascular endothelial growth factor (VEGF) is considered a significant contributor to the development of tumor vascular systems, as evidenced by the observed overexpression of VEGF in human tumor biopsies [16]. VEGF is a receptor tyrosine kinases (RTK)-interacting homodimeric disulfide-bound glycoprotein with a molecular weight of 34–45 kDa [17]. Angiogenesis under different conditions, including tumor pathogenesis, is regulated by *VEGF*, a gene family that includes several *VEGF* variants such as *VEGFA, B, C and D,* alongside placental growth factor *(PGF)*. Therefore, many studies have focused on *VEGFA* [18]. The VEGF receptor, VEGFR, consists of three domains: an extracellular domain for VEGF binding, a transmembrane domain, and an intracellular domain with tyrosine kinase activity that recruits various downstream molecules [19], resulting in the activation of downstream pathways, such as Ras/Raf/MEK/ERK [20] and PI3K/AKT/mTOR [21].

*Chen *et al*.* confirmed that intertumoral hypoxia is a notable trait of solid tumors, particularly liver cancer [22]. This condition stimulates VEGF production, subsequently leading to tumor angiogenesis [23]. The significant involvement of VEGF in liver cancer has been established in various studies. Approximately 5–7% of human liver cancer samples exhibit significant *VEGFA* gene amplification of [24]. Additionally, a correlation was observed between increased VEGF expression and reduced overall survival (OS) in a specific group of patients diagnosed with intrahepatic cholangiocarcinoma (ICC) [25]. *Muz *et al*.* reported the phenomenon that hypoxia-inducible factor (HIF) can be upregulated by hypoxia, which induces the activation of genes that promote angiogenesis, such as *VEGF* [26]. Subsequent research has confirmed that in a hypoxic microenvironment, the expression levels of VEGF and VEGFR are considerably upregulated, while the steadiness of HIF-1α diminishes, resulting in HIF-1α's attachment to the hypoxia response element (HRE), triggering angiogenesis [27]. A recent study has reported the involvement of lncRNAs in VEGF-related angiogenesis. A novel oncogenic lncRNA, PAARH, was identified in HCC, which facilitates the recruitment of HIF-1α to VEGF promoters by binding to HIF-1α. Consequently, this interaction upregulates VEGF expression and promotes angiogenesis [28]. Moreover, elevated levels of VEGF in CCA are linked to a higher likelihood of tumor advancement and reduced survival probabilities [29].

#### FGF

Fibroblast growth factors (FGFs) can be categorized into paracrine and endocrine FGFs [30]. Activation of the FGF signaling pathway occurs when FGFs bind to FGF receptors (FGFR1-4). This interaction induces dimerization, activation, and tyrosine phosphorylation of FGFR. Consequently, pathways such as Ras/Raf/MEK/ERK and PI3K/AKT/mTOR are activated as downstream response [31]. The FGF signaling controls a variety of cellular functions including survival, self-renewal, differentiation, metabolism, proliferation, epithelial mesenchymal transition (EMT), systemic angiogenesis, immunity in the microenvironment, and homeostasis [32, 33].

Research examining the impact of FGFR4-mediated signaling on the aggressive traits of HCC cells uncovered that FGFR4 expression was elevated in approximately 50% of acquired cases. Additionally, the upregulation of multiple FGF ligands suggests the potential involvement of the FGF pathway in HCC progression [34]. Increasing evidence suggests the significant involvement of FGFs in HCC development. *Kin *et al*.* reported the significant role of FGF2 as a potent mitogen in HCC development by targeting IIIc isotype of FGFR1, which was proved to promote tumor growth in hepatoma cell lines possibly by stimulating DNA synthesis [35]. *Pei *et al*.* confirmed that the upregulation of FGF8 stimulated cellular proliferation in human HCC cells. In addition, the researchers found that FGF8 not only promotes tumor cell growth, but also upregulates EGFR expression, leading to increased angiogenesis and resistance to EGFR inhibitors [36]. Additionally, *Paur *et al*.* demonstrated that FGF9 enhances the proliferation and invasiveness of HCC cells via FGFR3-IIIb/IIIc and simultaneously stimulates neo-angiogenesis and lymph angiogenesis [37]. Furthermore, *Raja *et al*.* found that the activation of FGF19/FGFR4 leads to the creation of a complex within HCC, consisting of FGF receptor substrate 2 (FRS2) and growth factor receptor-bound protein 2 (GRB2), which subsequently activates the MAPK/ERK and PI3K/AKT pathways [38].

Alterations or amplification of genes involved in the FGF signaling pathway can result in abnormal activation. Aberrant splicing of the FGFR3 transcript has been shown to promote HCC malignancy by modifying its ligand-binding domain, enabling self-activation via homodimerization and autophosphorylation, even in the absence of a ligand [39]. Additionally, *Greenman *et al*.* found that *FGFR4* mutations are associated with increased local growth and metastasis in HCC [40]. Additionally, the dysfunctional FGF signaling pathway has been verified as playing a role in the development of CCA. Notably, mutations in FGFR1 and FGFR3 are commonly observed in cases of CCA [41], while a previous study observed *FGFR4* overexpression in around 50% of all CCA cases, strongly indicating its carcinogenesis role [42].

#### TGF-β

The transforming growth factor-β (TGF-β) signaling pathway affects most cell types within human body. It plays a role in controlling a variety of cellular functions, including cell apoptosis, differentiation, growth, invasion, angiogenesis, production of the extracellular matrix (ECM), and the immune response. Additionally, it is crucial in the early stages of embryonic development and in maintaining the homeostasis of adult tissue [43]. Receptors of the TGF-β signaling pathway encompass two pairs of receptor serine/threonine kinases, namely the TGF-β I receptor (TGFβRI) and TGF-β II receptor (TGFβRII), which are universally expressed in all cell types [44]. TGF-β activates downstream pathways upon binding to receptor complexes, which can be categorized into SMAD and non-SMAD signaling pathways.

The activation of the SMAD signaling pathway occurs through the interaction of TGF-β with its receptors, which results in their phosphorylation. TGF-βRI recruits receptor-activated SMADs (R-SMADs), specifically SMAD2 and SMAD3. Phosphorylated R-SMADs dissociate from TGF-βRI and bind to SMAD4, forming a trimeric complex that translocates into the nucleus. This complex subsequently influences the transcription of specific genes through interactions with high-affinity DNA-binding transcription factors (TFs) and chromatin remodeling proteins (CRs) [45, 46].

In addition to SMAD signaling, TGF-β receptors in the liver can activate non-SMAD responses including the MAPK/ERK [47], PI3K/AKT/mTOR [48], and JAK/STAT3 signaling pathways [49].

Previous studies have established the influence of SMAD signaling on various processes, such as embryogenesis, adult tissue homeostasis [45], and malignant behaviors, such as migration, metastatic invasion, and EMT in tumorigenesis [50]. SMAD signaling strongly correlates with fibrogenesis [51]. The TGF-β pathway is essential for liver homeostasis and is involved in a wide range of physiological and pathological situations, including injury, inflammation, fibrosis, cirrhosis, and hepatocellular carcinoma [52].

The dual nature of the TGF-β signaling pathway in tumor development has led to what is known as the TGF-β paradox. The TGF-β signaling pathway exerts multiple effects on tumor cells, including apoptosis induction, inhibition of tumor cell proliferation, regulation of genomic instability, promotion of EMT, stimulation of neo-angiogenesis, immune damage, cell migration, and promotion of metastasis [53]. These effects ultimately contribute to tumor proliferation. This phenomenon can be attributed to the activation of two distinct sets of TGF-β response genes [54]. This phenomenon could also result from cells' capacity to bypass the cytostatic effects of TGF-β-SMAD signaling through mechanisms that are yet to be identified [55].

Multiple studies have demonstrated that the TGF-β signaling pathway exerts a tumor suppressor effect on the pathogenesis of HCC. A study investigating the relationship between TGF-β and cancer stem cells revealed that the absence of TGF-β signaling results in stem cell dysfunction, which triggers the onset of HCC [56]. Another study demonstrated that defective TGF-β pathway is crucial for CD133^+^ tumor-initiating stem-like cells (TICs) to induce HCC [57]. Research involving 147 patients with HCC (Hepatocellular Carcinoma) indicated that those exhibiting inactive TGF-β signaling pathways experienced reduced survival times in contrast to individuals with active TGF-β signaling. Approximately 38% of the 202 samples showed somatic mutations in at least one gene, resulting in disruption of the TGF-β signaling pathway [58].

Elevated TGF-β levels have been demonstrated to facilitate the carcinogenesis of liver cells. A previous study in mice demonstrated a strong link between excessive TGF-β expression and the formation of liver tumors [59]. *Abou-Shady *et al*.* found increased activation of TGF-β signaling in samples from human HCC, indicating its role in the onset and advancement of liver cancer [60]. Moreover, *Wu *et al*.* demonstrated that hepatoma-initiating cells may originate from hepatic progenitor cells through persistent TGF-β stimulation in cirrhotic liver [61]. These results suggest that the activated TGF-β signaling pathway may contribute to the pathogenesis of HCC. Additionally, *Lustri *et al*.* highlighted the involvement of the TGF-β signaling pathway in the progression of CCA [62]. Blocking TGF-β signaling with CX-4945 and LY2157299 demonstrated antitumor effects.

The TGF-β signaling pathway in HCC exhibits alterations in its ligands, receptors, and SMAD proteins. Individuals with HCC exhibit notably elevated levels of TGF-β compared to those with a healthy liver [52]. High TGF-β levels are closely linked to more advanced stages and unfavorable outcomes in patients with HCC [63]. In addition to the Ras/Raf/MEK/ERK and PI3K/AKT/mTOR signaling pathways, elevated TGF-β levels can also promote HCC development via a positive TGF-β/c-KIT feedback loop or TGF-β1/CD147 self-sustaining network [64, 65]. Additionally, *Bedossa *et al*.* suggested a link between a defect in TGF-βRII and the ability of TGF-β1 to bypass cell proliferation control in tumoral hepatocytes [66].

The dysregulation of SMAD protein was also found to be significant. *Yan *et al*.* observed a significant downregulation of the CXXC-type zinc-finger domain-containing protein (CXXC5) in HCC cells compared to that in normal liver tissues, which counteracted the inhibitory effect of histone deacetylase (HDAC1) on SMAD2/3. This consequently increased the activity of the TGF-β signaling pathway by inducing cell cycle arrest and apoptosis [67].

#### EGFR

The epidermal growth factor receptor (EGFR; also referred to as HER-1 or ErbB1) is a member of the tyrosine kinase receptor (TKR) family [68]. There are 7 types of ligands, including transforming growth factor-α (TGF-α), epiregulin (EREG), epidermal growth factor (EGF), amphiregulin (AR), betacellulin (BTC), epigen (EPGN), and heparin-binding EGF (HB-EGF) [69]. Under normal conditions, the EGFR signaling pathway regulates the proliferation and survival of epithelial cells, thereby promoting organogenesis and tissue repair [70–72]. Numerous studies have shown the critical role of EGFR signaling in the regeneration of liver tissue [73, 74]. *EGFR* is a frequently mutated oncogene that participates in liver tumorigenesis [75]. Activation of the EGFR signaling pathway upregulates downstream pathways, such as the Ras/Raf/MEK/ERK and PI3K/AKT/mTOR signaling pathways [76].

Evidence suggests that EGFR levels are significantly higher in human HCC tissues than those in normal tissues [77]. Additionally, there is an overexpression of various ligands such as TGF-α [78], EGF [79], HB-EGF [80], AR [81], and BTC [82]. Overexpression of EGFR or its ligands has been shown to have a significant pro-tumor effect. *Herbst *et al. demonstrated the significance of the EGFR signaling pathway in tumor cell differentiation, survival, and proliferation [83]. *Liu *et al*.* compared EGF expression between 97 HCC samples and 50 adjacent normal tissues. EGF levels were significantly elevated in HCC tissues, and the activation of EGF/EGFR signaling correlated with aggressive tumor characteristics and intrahepatic metastasis [79]. VersicanV1 in HCC has been found to interact with EGFR via an EGF-like motif. This interaction promotes HCC progression via the EGFR-PI3K-AKT signaling pathway [84]. Moreover, *Kou *et al*.* explored the function of the lncRNA NEAT1 in HCC and confirmed its elevated expression in HCC tissues, which inhibited hepatoma cell apoptosis and induced cell cycle progression via the EGFR pathway [85]. *Detarya *et al*.* identified *GALNT5* as a modulator of EGFR signaling in CCA, and its upregulation promotes CCA progression by inducing EGFR expression [86].

#### IGF

The insulin-like growth factor (IGF) system comprises several components, including specific ligands (IGF1 and 2), binding proteins (IGFBP, No.1–8), IGF receptors (type I and II), and IGFBP-specific proteases, despite its tissue specificity [87, 88]. IGF1 and IGF2 are named based on their structural homology and metabolic effects resembling those of insulin, which include higher growth-promoting activity [89, 90]. The liver is responsible for synthesizing IGF1, IGF2, and IGFBPs, which influence systemic metabolism and growth [91].

IGFs (endocrine, autocrine, and paracrine) and their receptors (IGF1R and IGF2R) have regulatory functions in the liver, particularly in hepatocyte progression, proliferation, and differentiation. These processes are crucial for the development, growth, and regeneration of the liver [92]. Dysregulation of the IGF signaling pathway may result in abnormal physiological processes in the liver [93].

IGFs and IGF-1R have been established as playing crucial roles in the development of liver cancer. Previous studies have reported a 20-fold upregulation in IGF-2 expression in transgenic HCC mice compared to the control group, suggesting its potential as an important initiating factor in HCC development [94]. *Mukherjee *et al*.* further confirmed the significance of IGF-2 in hepatocarcinogenesis in rats models [95]. *IGF2* overexpression in HCC was found to be associated with angiogenesis in vitro [96]. Furthermore, IGF-2 may promote HCC progression by inducing hypoglycemia [97].

IGF-1 has also been implicated in the development of HCC. *Weroha *et al*.* demonstrated that IGF1 promotes mitogenesis by stimulating DNA synthesis and inducing cyclin D1 expression in vitro [90]. Another study found that IGF1 promoted HCC cell growth and anabolism by inhibiting proteasome-mediated cathepsin B (CTSB) degradation [98].

Several studies have been centered on investigating the expression levels of IGF-1R. *Ngo *et al*.* found that IGF-1R signaling promotes HCC cell proliferation, activates cellular reprogramming, and induces cancer stemness, resulting in TKI refractoriness and tumor recurrence [99]. A study on miRNAs confirmed this finding, demonstrating that the downregulation of miR-448 expression in HCC promotes tumor progression through the reversal of IGF-1R-mediated glycolysis and cell survival inhibition [100].

#### c-Met

Hepatocyte growth factor (HGF), secreted by mesenchymal cells and expressed in hepatic stellate cells (HSCs), vascular endothelial cells (ECs), and Kupffer cells (KCs), functions as the sole ligand for the c-Met tyrosine kinase receptor [101–103]. Previous studies have revealed that the HGF/c-Met axis promotes survival and regeneration of the liver [104]. and plays a key role in liver growth and protection [105]. For example, a lack of HGF/c-Met signaling has been found to induce liver necrosis in animal models [106]. In contrast, an abnormally strong signal results in the proliferation of hepatocytes and hepatomegaly[107, 108]. HGF is implicated in lymph angiogenesis and neo-angiogenesis, and serves as an inducer of tumor growth, invasion, and metastasis [109, 110]. Dysregulation of the c-Met pathway can have negative consequences.

Currently, two modes of activation have been identified for the c-Met signaling pathway: canonical and non-canonical. The canonical c-Met signaling pathway involves HGF-mediated autophosphorylation of the c-Met cytoplasmic domain. This pathway subsequently activates downstream pathways, such as Ras/Raf/MEK/ERK [111] and PI3K/AKT [112]. The non-canonical c-Met signaling pathway is triggered by a range of factors, including the HGF analog des-γ-carboxy prothrombin (DCP) [113], crosstalk between activated EGFR and c-Met [114], and anoxia [115].

In a previous study, researchers observed that liver cell count reduction after the onset of liver disease promoted an upregulation of c-Met and/or HGF expression, which in turn stimulated hepatocyte proliferation, regeneration, and survival, ultimately leading to a delay in the development of liver disease. This process may also trigger or facilitate the progression of HCC [116].

Evidence suggests that c-Met plays a critical role in carcinogenesis of HCC. A clinical study revealed that c-Met expression is markedly elevated in HCC samples compared to adjacent non-tumorous liver tissues, ranging from 20 to 48% of cases [117]. Gene mutations are an important initiating factor in tumorigenesis. The first type is an aberration of the Met gene. Rearrangement of the OXR1-aMET variant has been identified in HCC and is thought to induce tumorigenesis via the non-canonical c-Met signaling pathway [118, 119]. Mutations in the genes encoding key proteins represent an additional category. For example, a mutation in the casitas B-cell lymphoma (Cbl)-binding domain of Y1003 is associated with carcinogenesis because the interaction between Cbl and Y1003 leads to c-Met ubiquitination and its subsequent removal from the cell surface, preventing persistent activation of c-Met [120, 121]. Met overexpression has been associated with CCA tumor development by activating downstream pathways [122, 123].

*Li *et al*.* found that c-Met can induce *VEGFA* in HCC, thereby promoting angiogenesis, which is vital for the survival of highly vascularized tumors, such as HCC [124]. Non-coding RNAs are involved in controlling the c-Met signaling pathway. *Bu *et al*.* found that the expression of *LINC00240* was upregulated in HCC cells, which promoted tumor cell viability, migration, and invasion through the LINC00240/miR-4465/HGF/c-Met axis [125]. Moreover, *Liu *et al*.* explored the role of miRNA-101 as a tumor suppressor in HCC and identified that miR-101 inhibited cell proliferation and migration by inhibiting c-Met signaling pathway [126].

#### PDGF

Within the PDGF (Platelet-Derived Growth Factor) system, there exist five kind of ligands in the form of dimers, including PDGF-AA, PDGF-BB, PDGF-CC, PDGF-DD, and PDGF-AB, alongside two receptors, PDGFR-α and PDGFR-β. PDGF-AA, PDGF-BB, PDGF-CC, and PDGF-AB have the capacity to bind to and activate PDGFR-α, while PDGF-BB and PDGF-DD uniquely bind to and activate PDGFR-β [127]. The PDGF system is crucial for cellular communication within the immune microenvironment [128], support the normal development of the nervous system [129], foster inflammatory conditions such as glomerulonephritis [130], contribute to organ fibrosis [131], and even relate to tumor carcinogenesis [132].

Given that HSCs are key players in the onset of liver fibrosis, considerable research efforts have been dedicated to understanding how the PDGF system promotes the fibrogenic activity of HSCs. Studies have revealed that the PDGF-B/PDGFR-β axis induces the aberrant activation of hepatic stellate cells, positioning it as a key initiator of liver fibrosis [133]. Additionally, the transmembrane receptor Neuropilin-1 (NRP1) has been identified as upregulated in the livers of mice with induced liver fibrosis. It promotes liver fibrosis through the action of several factors, including PDGF-BB on HSCs, facilitated by the stabilization of ubiquitin-specific peptidase 9X (USP9X) [134]. In a separate study using a mouse model for liver injury, researchers observed a significant upsurge in PDGFR-β levels alongside a notable reduction in miR-26b-5p. It was confirmed that miR-26b-5p negatively regulates PDGFR-β, thereby reversing liver fibrosis and angiogenesis [135]. Given the close association between liver fibrosis and liver cancer [136], there is no doubt that the PDGF system holds a significant position in the pathogenesis of liver cancer. *Xiao *et al*.* discovered the downregulation of *Xeroderma Pigmentosum D* (XPD, a subunit of Transcription Factor II H) and miR-29a-3p in the onset of HCC [137]. They further confirmed that miR-29a-3p, under the positive regulation of XPD, targets *Mdm2* (a negative regulator of P53) and the *PDGF-B* axis, inhibiting the progression of HCC. In a study related to the drug delivery system for liver cancer treatment, *Kaps *et al*.* identified that PDGFR-β, highly expressed in the cancer-associated fibroblasts (CAFs), can serve as a target for nanocarriers, thereby countering the tumor-promoting effects of CAFs [138].

### Upregulation of downstream receptor tyrosine kinase pathways

#### Ras/Raf/MEK/ERK signaling pathway

The Ras/Raf/MEK/ERK signaling pathway is typically upregulated by the aberrant activation of upstream growth factors and their receptors in HCC. Additionally, alteration of Ras/Raf/MEK/ERK signaling has also been implicated in HCC development [139].

The Ras/Raf/MEK/ERK signaling pathway comprises a cascade of tertiary MAPK enzymes, including upstream activators, specifically MAP3K, MAP2K, and MAPK. It involves activation of the small G protein Ras as MAP3K, MAPK/ERK kinase (MEK) as MAP2K, and ERK as MAPK [140]. The receptors involved in the Ras/Raf/MEK/ERK signaling pathway include PDGFR, FGFR, EGFR, VEGFR, IGFR, c-Met, and stem cell growth factor receptor (SCFR; also known as c-KIT) [141]. The engagement between growth factors and tyrosine kinase receptors triggers the phosphorylation and subsequent activation of these receptors, which results in downward signal transmission through Grb2/Shc/SOS molecules [142]. The initial step includes changing Ras from its inactive GDP-bound state to its active GTP-bound state, followed by recruitment of Raf to the cell membrane [143]. Subsequently, Raf, MEK, and ERK are sequentially activated. Once phosphorylated, ERK moves into the nucleus and activates two crucial transcription factors belonging to the AP-1 family, c-Jun and c-Fos [144], leading to transcription of target genes that play roles in cell cycle progression and other cellular processes related to cell growth [145].

The Ras/Raf/MEK/ERK signaling pathway is a key regulator of various physiological processes in cells, including growth, development, division, and apoptosis [140]. Dysregulation of the Ras/Raf/MEK/ERK signaling pathway typically results in atypical cellular behavior, including enhanced cell growth, dedifferentiation, survival, EMT, and migration, which contribute to carcinogenesis [144, 146, 147].

*Neuzillet *et al. found that Ras/Raf/MEK/ERK signaling was activated in approximately 50% of patients with early HCC and nearly all specimens from every case of advanced HCC [148]. This highlights the role of this pathway in HCC advancement. The mutation frequency of Ras/Raf/MEK/ERK signaling pathway components has been found to be relatively low In HCC [149]. For example, less than 5% of HCC instances exhibit mutations in Ras and Raf [150]. Frequent activation of the Ras/Raf/MEK/ERK signaling pathway in HCC can be attributed to various mechanisms, including downregulation of negative regulators and upregulation of positive regulators. Except for those two elements, multiple upstream growth factor receptors can somehow be activated in HCC [151]. For example, HCC recurrence has been found to be correlated with EGFR expression (47.1%), which in turn is correlated with TGF-α (45.7%) [152]. Considering that the activation of those upstream receptors has been discussed above, we mainly focus on the change of its regulators.

##### Downregulation of negative regulators

Raf kinase inhibitor protein (RKIP) naturally suppresses the MAPK/ERK signaling pathway. By competing for MEK phosphorylation, it prevents MEK activation through the separation of Raf-MEK complexes [153]. *Lee *et al*.* observed a heightened activation of the MAPK/ERK signaling pathway in HCC tissues, which they linked to reduced RKIP levels [154]. In addition, some tissues have demonstrated the function of dual-specificity phosphatase 1 (DUSP1), which serves to inhibit ERK activity [155]. Typically, DUSP1 is activated by ERK to establish a negative feedback loop in the MAPK/ERK signaling pathway, mediating its coordination [156]. *Calvisi *et al. found that unrestrained ERK activation leads to ubiquitin-mediated proteolysis of DUSP1 during the progression of HCC [155]. Ras GTPase-activating proteins (GAPs) are negative regulators that convert Ras from its GTP-bound activated state to its GDP-bound inactivated state. However, promoter hypermethylation and loss of heterozygosity (LOH) in some HCC cell lines downregulated RAS GAPs, such as *DAB2IP* and *RASAL1*, leading to an increase in active Ras, triggering the activation of subsequent pathways [144, 157]. RASSF1A and NORE1A, two Ras inhibitors, induce apoptosis by interacting with Ras [158]. *Calvisi *et al*.* observed active Ras in all 80 surgically resected HCC specimens in their study. In a further study, they found a reduction in at least one of the two Ras inhibitors in every sample, suggesting that downregulation is one of the requirements for HCC development [159]. The Sprouty protein negatively regulates the Ras/Raf/MEK/ERK signaling pathway by interfering with the GRB2/SOS complex formation and indirectly inhibiting the activation of Ras [160]. Sprouty2 (SPRY2) was downregulated in 31 HCC patients compared with their matched non-tumor tissues, indicating its critical tumor suppressor effects through the Ras/Raf/MEK/ERK signaling pathway [161].

##### Upregulation of positive regulators

Unlike RAS GAPs, Ras guanine nucleotide exchange factors (RAS GEFs) facilitate the exchange of GTP for GDP in Ras [162]. *Zhang *et al*.* observed that RASGRP1, which is part of the RAS GEFs family, exhibited higher expression levels in HCC compared to the adjacent non-cancerous tissue. This elevation was found to have a significant correlation with both the size and the stage of the tumor [163]. Furthermore, multivariate analysis indicated that overexpression of RASGRP1 independently predicts HCC progression risk [163]. miRNA-330-5P and lncRNA 01503 have been identified as positive regulators that induce HCC carcinogenesis through the Ras/Raf/MEK/ERK signaling pathway [164, 165]. Another study also identified the carcinogenic effect of the Ras/Raf/MEK/ERK signaling pathway in CCA [166]. Additionally, the antitumor effects of ^125^I [167] and miR-329 [168] were observed through the downregulation of MAPK signaling.

#### PI3K/AKT/mTOR signaling pathway

Oncogenes like *RAS* activate the PI3K/AKT/mTOR pathway, which, akin to the Ras/Raf/MEK/ERK pathway, operates downstream of growth factors [169]. The phosphatidylinositol 3-kinase (PI3K)/AKT (protein kinase B), the mammalian target of rapamycin (mTOR), and PI3K/AKT/mTOR pathways regulate various cellular processes, including growth, migration, survival, glucose and lipid metabolism, angiogenesis, inflammation, EMT, and dedifferentiation [170–172]. The PI3K/AKT/mTOR pathway, similar to the MAPK/ERK signaling pathway, is classified as the RTK pathway [141]. The interaction of ligands with RTKs results in activation of the PI3K/AKT/mTOR cascade through the phosphorylation and dimerization of each monomer [173]. PI3K is activated and catalyzes the conversion of phosphatidylinositol 4,5-bisphosphate (PIP2) to phosphatidylinositol 3,4,5-triphosphate (PIP3), which subsequently activates AKT [174]. Activated AKT phosphorylates cytoplasmic and nuclear target proteins, including mTOR, to exert its effects [175]. The PI3K/AKT/mTOR signaling pathway is crucial for maintaining cell survival and promoting proliferation [176]. Therefore, dysregulation of this pathway in pathological conditions such as cancer causes abnormal cell survival [177].

Liver cancer is associated with mutations and dysregulation of the PI3K/AKT/mTOR signaling pathway. PTEN is a negative regulator of PI3K. However, *Kudo *et al*.* reported that up to 53% of patients with HCC exhibited *PTEN* gene loss, resulting in elevated PIP3 levels. Thus, overactivation of the PI3/AKT/mTOR pathway inhibits apoptosis and promotes HCC tumorigenesis [178, 179]. *Luo *et al*.* initially elucidated the role of YTH N6-methyladenosine RNA-binding protein 1 (YTHDF1) in HCC. Found in higher levels within HCC, its association with the severity of tumor stage and the enhancement of HCC cell growth is through stimulating the PI3K/AKT/mTOR pathway [180]. Later, *Pu *et al*.* reported the presence of valosin-containing protein (VCP) in HCC patients with advanced TNM stages and poor prognosis. The researchers also confirmed that VCP overexpression in HCC cells caused protumor effects via the PI3K/AKT/mTOR pathway in vitro and in vivo [181]. *Hao *et al*.* found overexpression of serine/threonine kinase 39 (STK39) in CCA, which activated PI3K/AKT signaling and promoted tumor growth [182].

The PI3K/AKT/mTOR signaling pathway-related lncRNAs also contribute to the pathogenesis of HCC. *Wu *et al*.* identified a group of PI3K/AKT/mTOR signaling-related lncRNAs implicated in HCC, and elucidated their roles in HCC development [183]. Later, *Liu *et al*.* observed an increase in lncRNA-PICSAR levels in HCC samples, which promoted cell cycle progression, colony formation, and inhibited HCC cell apoptosis. Subsequent studies revealed that PI3K/AKT/mTOR activation by lncRNA-PICSAR causes oncogenesis, leading to HCC [184]. *Song *et al. explored the underlying mechanism and effects of lncRNA RHPN1 antisense RNA 1 (RHPN1-AS1) on HCC. The lncRNA RHPN1-AS1 is upregulated in HCC tissues and cells and is associated with the malignant phenotype in HCCLM3 and MHCC97-H cells by targeting miR-7-5p and activating the PI3K/AKT/mTOR pathway [185].

## Growth factor receptor-independent signaling pathways in liver cancer

The signaling pathways mentioned in this segment activate independently of growth factor receptor ligand binding. We explore the composition of the Wnt-β-catenin, JAK/STAT, Hedgehog, Hippo, Notch, and NF-κB signaling pathways and their regulatory roles in the progression of liver cancer. Interestingly, under certain circumstances, some pathways exhibit crosstalk [186]. But to elucidate their functions clearly, we offer a comprehensive review of each one.

### Wnt-β-catenin signaling pathway

Wnt-β-catenin signaling abnormalities in HCC progression are induced by genetic alterations of Wnt-β-catenin signaling components such as *CTNNB1* and *AXIN*, hypoxic microenvironment, and chronic inflammation resulting from HBV/HCV infection [187]. The Wnt signaling pathway is categorized into non-canonical (independent of T-cell factor/lymphoid enhancer-binding factor, known as TCF/LEF) and canonical (β-catenin-dependent) pathways [188] based on the three distinct domains of DVLs: the amino-terminal DIX domain, central PDZ domain, and carboxyl-terminal DEP domain, which alter the downstream pathway of Wnt signaling [189]. The noncanonical pathways of Wnt signaling encompass the Wnt planar cell polarity and the Wnt/Ca2 + pathway, regulate cell polarity and migration [190]. Therefore, we primarily focused on the canonical Wnt signaling pathway, also known as the Wnt-β-catenin signaling pathway. The Wnt-β-catenin signaling pathway consists of several components. The membrane receptor consists of the extracellular ligand Wnt protein, a seven-channel transmembrane co-receptor Frizzled (FZD), and the single-channel transmembrane co-receptor (LDL receptor-associated protein 5/6, LRP5/6) [191]. The cytoplasmic portion mainly includes β-catenin, disheveled protein (DVL), glycogen synthase kinase-3β (GSK-3β), scaffold protein Axin, adenomatous polyposis coli protein (APC), and casein kinase 1γ (CK1γ), while the core portion primarily contains β-catenin, β-catenin-TCF/LEF) family members, and β-catenin downstream target genes such as MMPs and c-Myc [190].

**Fig. 3:**
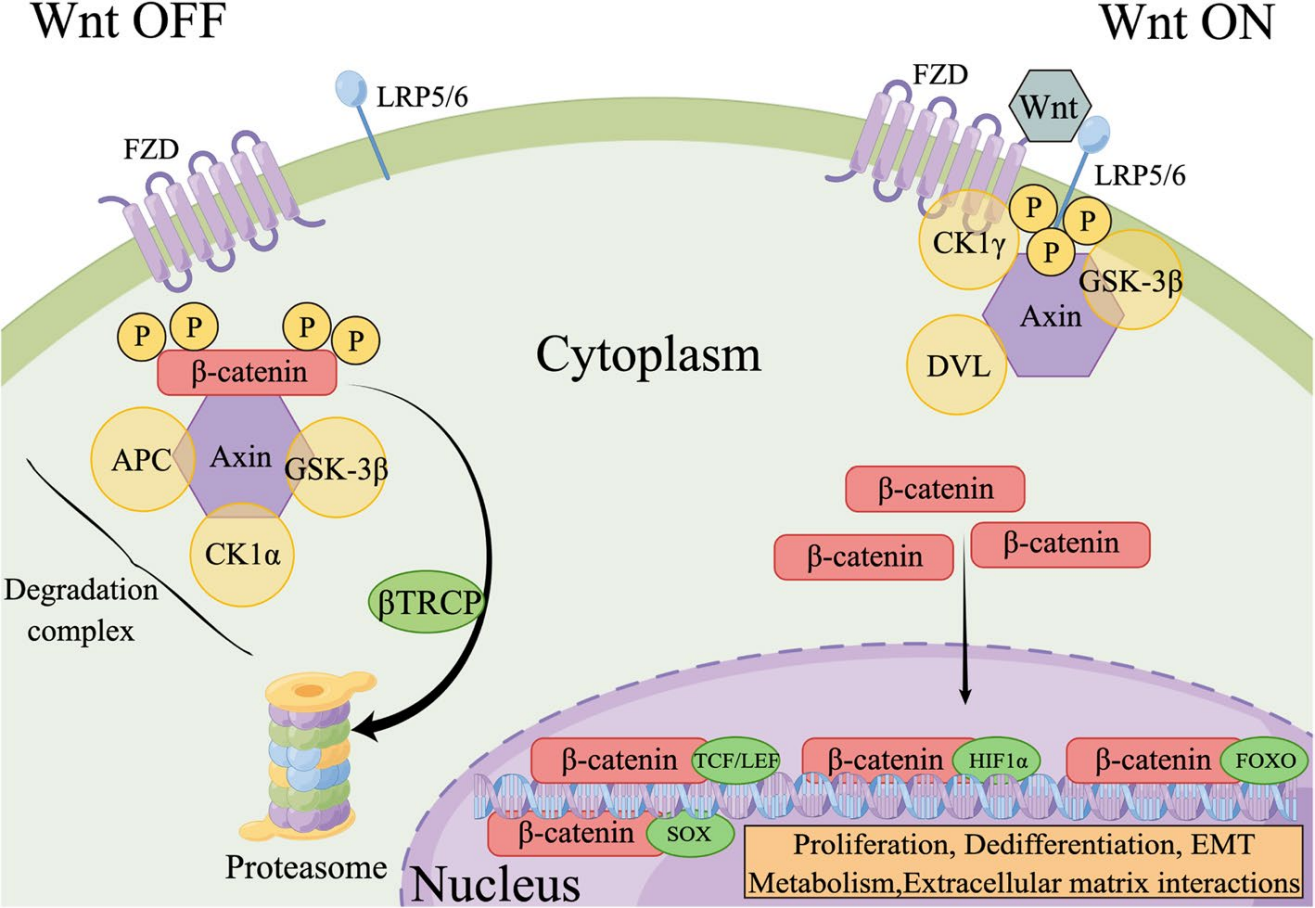
Wnt-β-catenin signaling pathway. When Wnt signaling is inactive (Wnt OFF), β-catenin within the cytoplasm undergoes phosphorylation by a degradation complex, which subsequently leads to its breakdown via the proteasome. Conversely, in the presence of Wnt ligands (Wnt ON), the interaction between the FZD and LRP5/6 receptors is initiated, followed by the phosphorylation of LRP6. This event attracts GSK-3β, CK1γ, and DVL, disrupting the Axin-led phosphorylation of β-catenin. Consequently, β-catenin accumulates in the cytoplasm and then relocates to the nucleus, where it regulates the transcription of specific genes. By figdraw

The Wnt-β-catenin signaling pathway primarily regulates various cellular processes, including proliferation [190], dedifferentiation, EMT, extracellular matrix interactions, inflammation, and metabolism [192]. It is also associated with liver cell differentiation, repair, and homeostasis [193]. Without Wnt ligands, cellular homeostasis is regulated by the β-catenin degradation complex, consisting of Axin, APC, GSK-3β, CK1α [194, 195]. The degradation complex sequentially phosphorylates β-catenin at four sites, leading to its subsequent degradation by the E3 ubiquitin ligase subunit, βTRCP-mediated proteasome [196]. Activation of the Wnt-β-catenin signaling pathway begins with the interaction between Wnt ligands and the binding domain of FZD, which triggers the formation of the FZD-LRP receptor complex [191]. Upon LRP6 phosphorylation, GSK-3β, CK1γ, and DVL bind and prevent the phosphorylation of β-catenin by Axin, thus blocking its degradation [193]. Finally, stabilized β-catenin is able to translocate to the nucleus and activates target genes through transcription factors, such as TCF/LEF [197], HIF1α [198], FOXO [199], and SOX [200] (Fig. 3). Therefore, dysregulation of this pathway is associated with the onset and progression of liver diseases including cancer [201].

One hallmark of liver cancer is the activation of the Wnt-β-catenin signaling pathway through somatic genetic and nongenetic events [202]. *Lee *et al*.* reported that *CTNNB1* and *AXIN1* mutations have a prevalence of 30% and 11%, respectively, making them the most frequently observed somatic genetic event [203]. *CTNNB1*, which encodes β-catenin, is one of the most mutated genes in the Wnt-β-catenin signaling pathway [203, 204]. Gain-of-function mutations in *CTNNB1* in HCC inhibit β-catenin phosphorylation and subsequent breakdown, leading to the activation of the Wnt-β-catenin signaling pathway, which then enhances cellular growth, and movement [201, 205]. Similarly, a loss-of-function mutation in *AXIN1* disrupts Axin in the β-catenin degradation complex, causing dysregulation of the Wnt-β-catenin signaling pathway, thereby disrupting cellular homeostasis [187]. Additionally, mutations in *CTNNB1* and *AXIN1* have similar pro-tumor effects on CCA, inducing cell proliferation and inhibiting apoptosis [206, 207].

Growing evidence suggests that nongenetic events contribute to the development of liver cancer. The excessive binding of Wnt ligands to receptors is of great significance in the development of HCC. A previous study revealed that SFRP1 promoter methylation, which encodes a Wnt signaling antagonist, is present in 75% of hepatoma cell lines and 48.2% of primary HCCs. This methylation results in the downregulation of *SFRP1* expression and promotes Wnt ligand-receptor interaction [208]. In addition, HCC tumor cells and inflammation-activated macrophages overexpress Wnt ligands, activating the Wnt-β-catenin pathway [209, 210]. A similar phenomenon was observed in the CCA. *Boulter *et al*.* found that inflammatory macrophages in CCA stroma express the WNT7B ligand, maintaining a high WNT-microenvironment [211].

*Fu *et al*.* found that linc00210 promotes the self-renewal and propagation of liver TICs in HCC tissues through the Wnt-β-catenin signaling pathway, specifically through the interaction between β-catenin and TCF/LEF components [212]. Another study investigating early recurrence in HCC has revealed the oncogenic role of AKIP1, a binding partner of β-catenin. AKIP1 not only impedes β-catenin degradation but also facilitates the recruitment of cyclic AMP response element-binding protein (CBP) through β-catenin, consequently activating downstream transcription of the Wnt-β-catenin pathway. Thus, the AKIP1/β-catenin/CBP axis is implicated in the recurrence of HCC [213]. Moreover, *Song *et al*.* showed that transcription factor activating enhancer-binding protein 4 (TFAP4) directly interacts with the promoters of DVL1 and LEF1 to improve HCC cell tumorigenicity via the Wnt-β-catenin signaling [214]. Recently, TMEM9 has been found to be highly expressed in cancer cells. This leads to activation of the Wnt-β-catenin signaling pathway. TMEM9 directly bind to the subunit of v-ATPase and its accessory protein, resulting in vesicular acidification and the lysosomal APC degradation [215], *Jung *et al*.* further confirmed that TMEM9-v-ATPase induced nuclear translocation of β-catenin by downregulating APC, resulting in hepatic tumorigenesis [216].

### JAK/STAT signaling pathway

The progression of HCC is linked to the enhanced activity of the JAK/STAT signaling pathway in the liver, a process affected by the epigenetic suppression of *SOCS* genes, inflammatory and oxidative stress, and growth factor stimulation [217]. IL-6, a STAT3-activating cytokine, is overexpressed in liver diseases including HCC [218]. The JAK/STAT signaling pathway encompasses four components: extracellular ligands (such as cytokines from the IL-2, IL-3, and IL-6 families, as well as interferon) [219]; transmembrane receptors; cytoplasmic components (including Janus Kinases or JAKs); a family of signal transducers and activators of transcription (STATs), and core portions (comprising dimer-forming STATs and their downstream target genes such as *BIRC5, CCND1*, and *MCL1*) [220, 221].

**Fig. 4:**
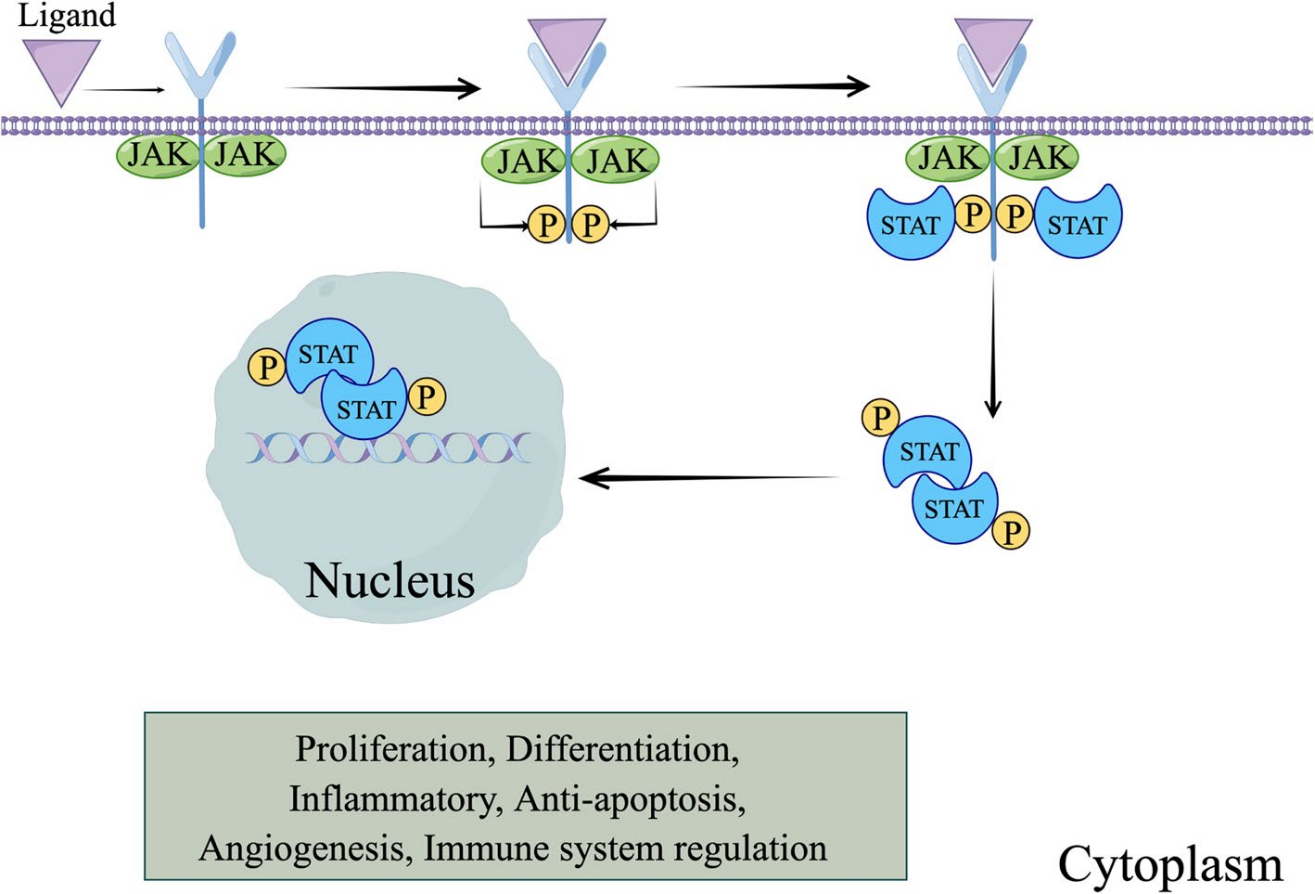
JAK/STAT signaling pathway. Ligand-receptor interaction triggers the phosphorylation of the receptor's cytoplasmic domain via JAK activation, establishing docking points for STAT attachment and its subsequent phosphorylation by JAK. These phosphorylated STAT molecules then dimerize within the cytoplasm and migrate to the nucleus, where they direct the transcription of specific genes. By figdraw

The JAK/STAT signaling pathway regulates various cellular functions, including differentiation, proliferation, immune system regulation, and inflammatory responses [222]. Binding with a ligand induces a structural modification in the receptor, leading to activation of JAKs in the cytoplasmic tail. These JAKs phosphorylate particular tyrosine sites on the cytoplasmic end of the receptor, forming attachment points for STATs to bind, followed by phosphorylation of STATs and the formation of dimers. Subsequently, it translocates to the nucleus, where it recognizes and interacts with specific promoter sequences, resulting in the transcription of target genes (Fig. 4) [220].

JAK/STAT3 signaling pathway is closely associated with HCC carcinogenesis. *He *et al*.* identified activation of JAK/STAT3 signaling in over 60% of HCC specimens [217]. During the development of liver cancer, the JAK/STAT3 pathway can be activated via mechanisms that both require and do not require ligands. Ligand-related pathway activation mainly includes two scenarios, the first of which is ligand overexpression. IL-6/STAT3 signaling may influence the initiation, survival, and progression of HCC [223, 224]. This phenomenon arises from the interaction between STAT3 and nuclear factor kappa B (NF-κB) pathways. In the HCC microenvironment, IL-6 is released by the activated NF-κB pathway, and eventually, the NF-κB/IL-6/STAT3 pathway exerts a pro-tumor effect in inflammation-associated tumors, such as HCC [225, 226]. The second mechanism involves downregulation or loss of JAK/STAT inhibitors. In HCC samples, downregulation of at least two inhibitors, CIS and SOCS, was observed in all 80 samples in a study, which can be attributed to promoter hypermethylation and LOH of JAK/STATS inhibitors [159]. Somatic genetic events can lead to the activation of ligand-independent pathways. *Kan *et al*.* reported that 9% of patients with hepatitis B-associated HCC had gain-of-function *JAK1* mutations. It stimulates JAK1 and STAT3 phosphorylation, and increases cell proliferation in the absence of ligands in vitro [227]. *Sia *et al. identified STAT3 activation as a characteristic feature of ICC inflammation (38% of ICCs) [228]. *Sun *et al. confirmed the activation of JAK/STAT signaling during the transformation of human induced hepatocytes (hiHeps) into ICCs, which could be inhibited by suppressing JAK/STAT and relevant signaling [229].

The mechanisms by which JAK/STAT3 drive HCC have been investigated. Activated STAT3 upregulates anti-apoptotic target genes (*BCL2*, *BCL2L1*, *BIRC5*, *CCND1*, and *MCL1*) [230–232], and downregulates pro-apoptotic proteins (TP53, BAX, and CHOP) [233, 234], thereby promoting cell survival. In contrast, STAT3 promotes angiogenesis, thereby supporting tumor progression and metastasis. *Liu *et al*.* validated the significance of the STAT3/HIF-1α/VEGF signaling pathway in HepG2 cells, highlighting its crucial role in angiogenesis and proliferation [235]. In addition, activated STAT3 contributes to immune escape. *Yin *et al*.* demonstrated that macrophages are capable of transitioning from an anti-cancer M1 type to a cancer-promoting M2 type when induced by IL-4, which promotes the proliferation, invasion, and migration of HCC cells [236]. *Bi *et al*.* demonstrated that STAT3 improves cellular energy metabolism. Their study revealed that STAT3 regulates the expression of PKM2, which is a key enzyme in the Warburg effect, to meet the energy needs for HCC cell proliferation [237].

### Hedgehog signaling pathway

Chronic liver damage arising from alcohol consumption, viral infection, and obesity triggers the activation of Hedgehog signaling, thereby promoting HCC tumorigenesis [238]. The Hedgehog pathway is a signaling mechanism preserved through evolution that relays information from the cell membrane to the nucleus [239]. Hedgehog (Hh) proteins, a class of secreted signaling proteins, play a crucial role in the development, maintenance, and repair of tissues and organs, including the liver [240].

**Fig. 5:**
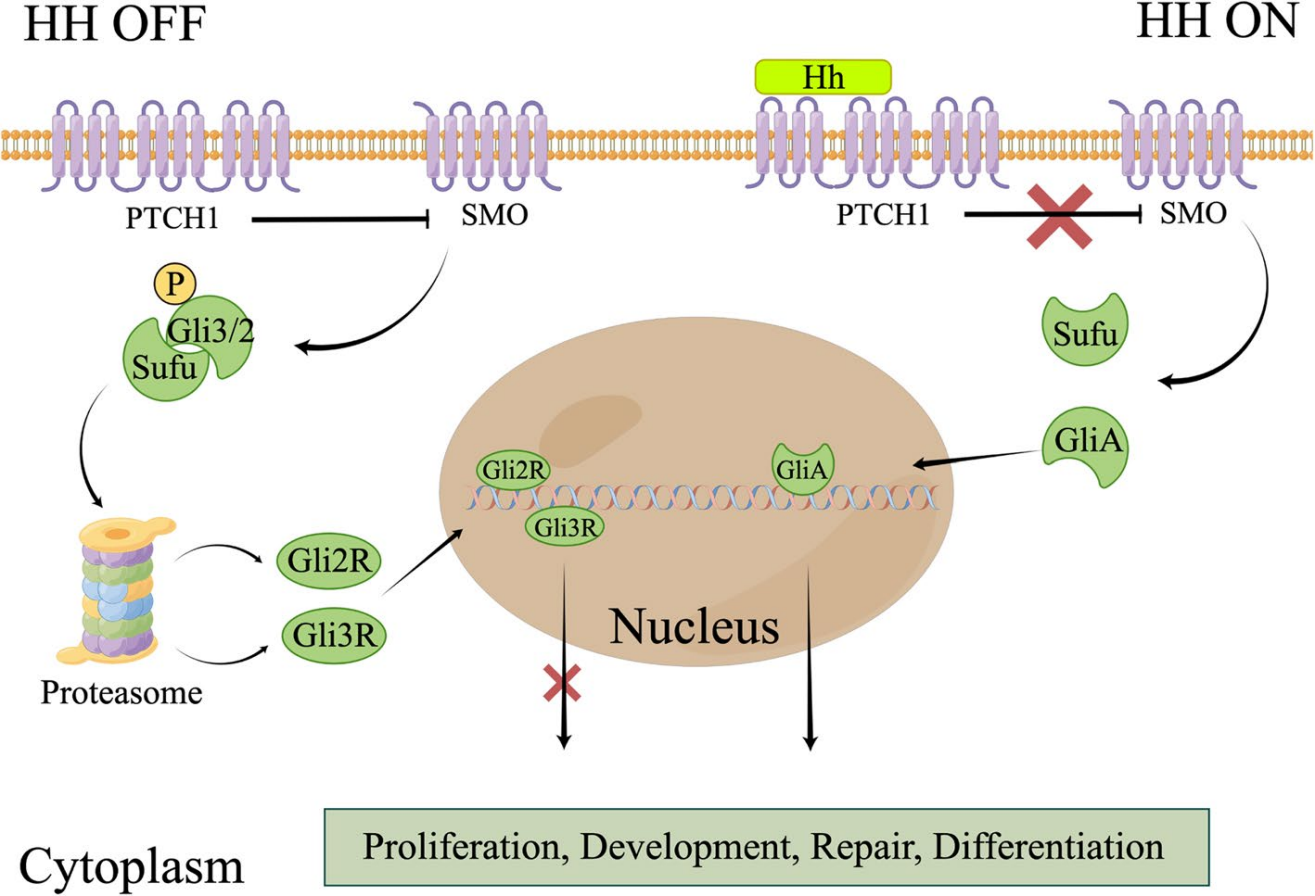
Canonical Hedgehog signaling pathway. In the absence of extracellular Hedgehog ligands (HH OFF), SMO is suppressed by PTCH1, leading to Gli2/3 phosphorylation by Sufu. This phosphorylation facilitates the proteasome-mediated partial degradation of Gli2/3. Subsequently, the repressor forms of Gli3 and Gli2 (Gli3/2R) are transported into the nucleus to repress the transcription of specific genes. When Hedgehog ligands are present (HH ON), their binding to PTCH1 lifts PTCH1's inhibition on SMO, enabling the activation of Gli proteins (GliA). GliA then enters the nucleus to promote the transcription of target genes. By figdraw

The canonical Hedgehog signaling pathway is composed of secretory ligands (Sonic, Indian, and Desert), 12-pass transmembrane protein patched 1 (PTCH1), heptahelical transmembrane G-protein-coupled receptor smoothened (SMO), and transcription factor GLI proteins (GLI1, GLI2, and GLI3) [241]. In the absence of ligands, PTCH1 inhibits SMO activation, resulting in recruitment of the suppressor of fused (Sufu) for inactivation of Gli3/2 [242]. Gli2 and Gli3 undergo phosphorylation, resulting in the formation of binding sites for E3 ubiquitin ligase βTRCP. The creation of the inhibitory forms Gli3/2R occurs through partial proteasome degradation, after which they move to the nucleus to repress the transcription of specific genes [243]. In the presence of Hh, it specifically binds to PTCH1, which removes its inhibition of SMO, leading to cleavage and release of the active form of Gli (GliA). Finally, GliA migrates to the nucleus to activate the transcription of target genes [244]. Moreover, accumulating evidence has shown SMO-independent stimulation of Gli activation, which is called the non-canonical Hedgehog signaling pathway [245]. We focused on the canonical Hedgehog pathway because of its interaction with other signaling pathways, including Ras/Raf/MEK/ERK [246] and PI3K/AKT/mTOR [247] (Fig. 5).

*Li *et al*.* observed that the Hh signaling pathway was typically inactive or weakly active in adult organs under normal conditions, unless stimulated by trauma [248]. In the adult liver, Hh ligands are abundantly expressed in various cell types, including Kupffer cells, progenitor cells, endothelial cells, activated HSCs, injured hepatocytes, and natural killer cells. Under such conditions, the Hh signaling pathway is activated to facilitate tissue repair [238]. The transient activation of the Hh signaling pathway during embryogenesis suggests its essential role in the differentiation and maturation of hepatic progenitor cells [249]. In summary, the Hh signaling pathway is crucial for liver development and repair, suggesting that its dysregulation may contribute to cancer development [250].

HCC carcinogenesis is associated with the Hh signaling pathway. *Efroni *et al*.* investigated sonic hedgehog (SHH) pathway expression in tumors and tumor-adjacent human HCC tissues. They revealed that the SHH pathway is frequently activated in tumor samples, suggesting a potential direct association with HCC development [251]. Later, *Cai *et al*.* found that increased SHH signaling promotes cell proliferation by facilitating the G2/M transition, which eventually results in the pathogenesis of liver cancer [252].

Ligand overexpression activates the Hh signaling pathway. In a previous study, SHH expression was detected in 60% of HCC samples [253]. Another study found elevated SHH levels in 15 of 21 HCC samples and all 19 CCA samples [254]. The biological significance of alterations in the Hh signaling composition in CCA remains unknown [255].

Changes in other elements of the Hh signaling pathway, besides ligands, have been established to have associations with the advancement of HCC. *Sicklick *et al. observed the overexpression of the *SMO* proto-oncogene in HCC. There was a noticeable positive relationship between the dimensions of the tumor and the ratio of SMO-to-Ptc mRNA expressions [256]. *Zeng *et al*.* suggested that PTCH-1, SHH, and GLI-1 mRNAs were highly expressed in over 98% of the selected HCC samples. Additionally, all HCC samples exhibited elevated PTCH-1 and GLI-1 expression compared to that in non-neoplastic liver tissues. This increased expression is strongly associated with an increased risk of recurrence and shorter OS [257].

### Hippo signaling pathway

Downregulation of the Hippo signaling pathway plays a role in shaping the immune microenvironment by regulating macrophage infiltration and tumor-associated macrophage (TAM) differentiation, contributing to the development of inflammation-induced cancer [186, 258]. Research has verified the Hippo signaling pathway's high degree of conservation across mammalian species, and it controls the growth and homeostasis of tissues and organs. Importantly, its dysregulation may lead to cancer [259–261].

**Fig. 6:**
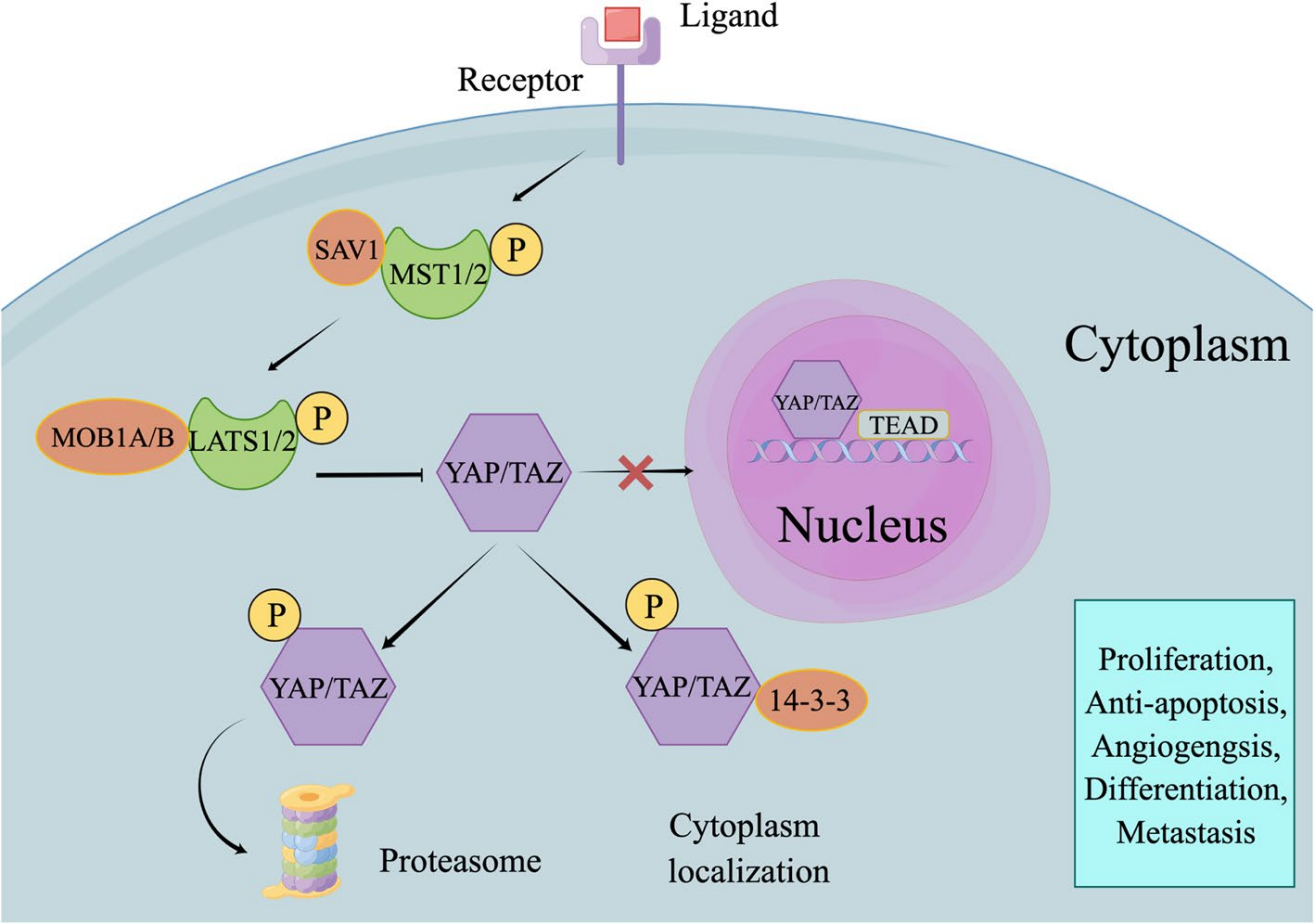
Hippo signaling pathway. Following the binding of specific ligands to their receptors, there is a sequential phosphorylation of MST1/2 and LATS1/2. Additionally, the presence of SAV1 and MOB1A/B enhances their activities. Notably, the phosphorylation of LATS1/2 leads to the phosphorylation and inactivation of YAP/TAZ, resulting in their retention in the cytoplasm or their degradation via the proteasome. Conversely, YAP/TAZ migrate to the nucleus to influence the transcription of target genes only when Hippo signaling is diminished. By figdraw

In mammals, the Hippo signaling pathway is comprised of kinase cascades and downstream effector molecules. Kinase cascades include mammalian STE20 like kinase 1/2 (MST1/2), the mitogen-activated protein kinase kinase kinase kinase (MAP4K) family, and large tumor suppressor kinase 1/2 (LATS1/2). The primary kinase cascades are MST1/2 and LATS1/2, the effects of which are enhanced through the involvement of adaptor proteins SAV1 and MOB1A/B following activation and phosphorylation [262]. Downstream effector molecules consist of Yes associated protein 1 (YAP1 or YAP)/WW domain containing transcription regulator 1 (WWTR1 or TAZ) transcription coactivators and DNA-binding protein TEA domain transcription factor 1/2/3/4 (TEAD1/2/3/4) [263]. Hippo kinase cascades have been found to inhibit transcription modules and impede the development of cells and tissues [263]. Upstream signals phosphorylate MST1/2 and LATS1/2 kinases, leading to the inactivation and cytoplasmic localization of YAP/TAZ. This occurs through their binding to 14–3-3 protein or proteasomal degradation [263]. Diminished activity in the Hippo pathway results in the nuclear translocation of YAP/TAZ and binds to TEAD1/2/3/4 as a substrate. This interaction facilitates the binding of TEAD1/2/3/4 to DNA, leading to regulation of target gene transcription (Fig. 6) [264, 265].

There is increasing evidence linking the Hippo signaling pathway to hepatocarcinogenesis. *Perra *et al. used a cancer-induced rat model that required YAP activation [266]. *Weiler *et al*.* investigated the relationship between the Hippo signaling pathway, chromosome instability (CIN), and HCC in mice and humans. They found that the activation of *YAP* contributes to CIN, leading to HCC development [267]. Another study using a mouse model demonstrated that *YAP* overexpression is required for the development of HCC driven by *c-Myc* and *AKT1* [268]. *Han *et al*.* found that the expression rates of *YAP*, *TAZ*, and their target gene *AREG* were 69.2%, 66.7%, and 61.5%, respectively. YAP has the potential to serve as an independent prognostic indicator, whereas serum AREG may function as a serological biomarker for HCC [269]. Another clinical trial demonstrated that hypoxia induces the translocation of YAP to the nucleus, where it interacts with HIF-1α. This interaction leads to the initiation of *PKM2* transcription, which in turn promotes glycolysis in HCC cells. However, this effect was reversed by *YAP* knock out [270]. Besides, *Chen *et al*.* found that the Hippo signaling pathway in HCC can be suppressed by the highly expressed cytoskeletal proteins alpha actinins (ACTNs), which exerted a protumor effect [271].

Similar effects of the Hippo signaling pathway have also been observed in other types of liver cancer. *Marti *et al*.* proved evidence of the tumorigenic role of YAP, since YAP facilitated angiogenesis and proliferation in CCA cells through its interaction with other regulatory molecules [272]. *Pei *et al*.* observed a significant positive correlation between nuclear YAP (nYAP) levels and various clinical factors such as TNM stage, histological differentiation, poor prognosis, and metastasis in CCA [273]. Regarding hepatoblastoma (HB), *Lu *et al*.* found elevated expression of YAP in HB patients compared to that in individuals without HB. The attachment of the YAP/TEAD4 transcription factor complex to the CTNNB1 promoter area stimulates the expression of β-catenin and cellular proliferation [274]. Moreover, *Liu *et al*.* reported that YAP/TAZ activates the mTOR complex 1 (mTORC1), which in turn stimulates HB development via the amino acid transporter SLC38A1 [275]. These findings also identified interactions between Hippo signaling and other pathways, underscoring the importance of focusing on pathway interplay.

### Notch signaling pathway

**Fig. 7:**
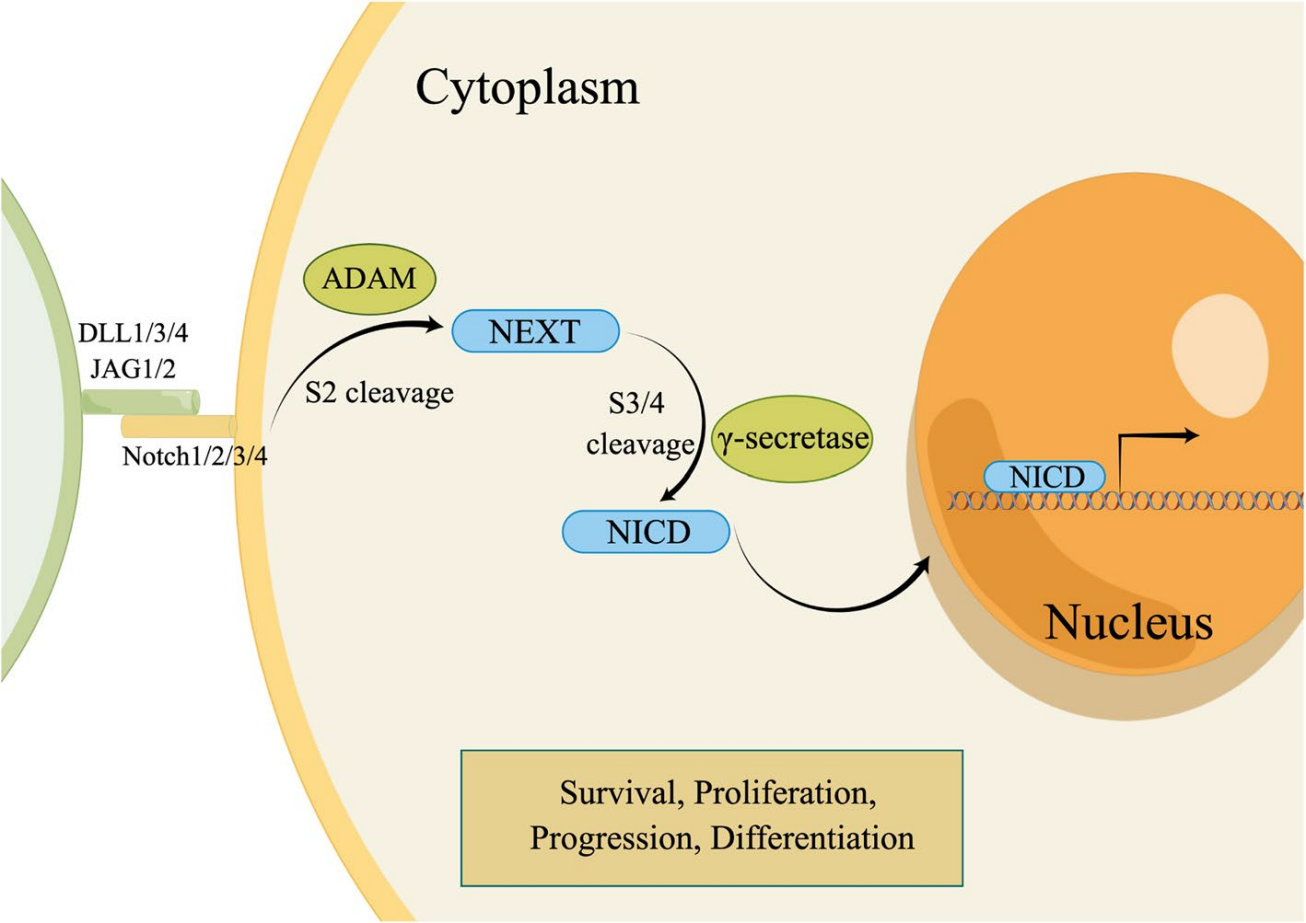
Canonical Notch signaling pathway. Cell-to-cell contact facilitates the interaction between receptors and ligands, leading to the endocytosis of Notch. The initial cleavage at site 2 (S2) by the ADAM generates the NEXT fragment, which is a substrate for further cleavage by the γ-secretase complex from site 3 (S3) to site 4 (S4). This process ultimately releases the Notch Intracellular Domain (NICD) fragment, which then travels to the nucleus to modulate the transcription of specific target genes. By figdraw

The Notch signaling pathway is closely associated with the HCC microenvironment. DLL4, a Notch signaling ligand, has been shown to induce an inflammatory response in macrophages [276], which leads to their differentiation into TAMs and the subsequent release of pro-tumorigenic cytokines [277]. The Notch signaling pathway is a highly conserved intercellular signaling pathway that plays a role in organ formation, tissue repair [278], cell differentiation, survival, and apoptosis [279]. Currently, four types of Notch receptors (Notch 1–4) and five Notch ligands (delta-like ligand 1/DLL1, delta-like ligand 3/DLL3, delta-like ligand 4 /DLL4, Jagged-1/JAG1, and Jagged-2/JAG2) have been identified in humans [280, 281]. There are two forms of the Notch signaling pathway: the canonical pathway, which is dependent on ligands, and the non-canonical pathway, which operates independently of ligands. In the canonical Notch signaling pathway, ligands and receptors engage through direct intercellular contact. This interaction triggers endocytosis and cleavage of Notch by ADAM (a disintegrin and metal-loprotease), a protease enzyme, at site 2 (S2). This cleavage generates a membrane-anchored Notch extracellular truncation (NEXT) fragment, which serves as a substrate for the γ-secretase complex. γ-secretase cleaves NEXT from site 3(S3) to site 4(S4), resulting in release of the Notch intracellular domain (NICD). Finally, NICD is transported to the nucleus as a transcription factor that regulates target gene expression [169, 278, 282, 283]. In addition to ligand binding, certain Notch receptors undergo endocytosis for degradation in lysosomes, or activation in endosomes for renewal [284, 285]. The focus of our discussion is primarily on the canonical Notch signaling pathway, as the non-canonical pathway interacts with other signaling pathways (Fig. 7).

The Notch signaling pathway's dysfunction has been implicated in the development of various forms of cancer [286]. Similarly, the Notch signaling pathway has been implicated in liver tumorigenesis. In 2009, a study reported the initial use of Notch1 signaling downregulation as a therapeutic approach for the treatment of patients with HCC [287]. At that time, there was limited knowledge regarding the involvement of the Notch signaling pathway in liver cancer. Several years later, *Razumilava *et al*.* conducted a study on the Notch signaling pathway in HCC. Activated Notch signaling was observed in about 30% of HCC specimens. Additionally, activation of Notch signaling promotes the development of liver tumors in mouse models [288]. Subsequently, *Ahn *et al*.* reported that abnormal Notch signaling promotes HCC development. Notch1 and Notch4 have been identified as potential biomarkers associated with shorter survival in patients with HCC [289]. Abnormal expression of *NOTCH1* and *NOTCH4* has been found to be correlated with ICC [290]. *Wu *et al*.* identified that overexpression of *NOTCH1* is linked to a larger tumor size, while upregulation of *NOTCH4* is linked to elevated serum CA125 concentrations. *Zhao *et al*.* assessed the expression of Notch ligands and receptors in HCC. In 370 HCC samples, the expression levels of Notch 1, Notch 2, Notch 3, Notch 4, DLL4, JAG1, and JAG2 were markedly greater in tumor samples compared to surrounding non-tumorous tissue. Additionally, higher levels of DLL3 and DLL4 are associated with poor OS, and DLL3 levels are correlated with TNM stage [291]. *Zhu *et al*.* confirmed that Notch activity is increased in liver cancers, and they demonstrated that aberrant and sustained Notch signaling activity induces cancer progression, thus contributing to carcinogenesis in animal models [292].

These studies have highlighted the significance of abnormal Notch ligands and receptors in HCC tumorigenesis. Other studies have also validated the involvement of Notch in the pathogenesis of HCC. *Kongkavitoon *et al*.* conducted a study on HBV, a major significant contributor to HCC. These results demonstrated that the HBx-DLL4-Notch1 axis plays a role in promoting tumor growth by maintaining HCC cell survival [293]. Additionally, a previous study showed high expression of the small chromosome maintenance protein, *MCM6*, in HCC [294]. *Liu *et al*.* further confirmed the pro-tumor role of *MCM6* in promoting HCC cell proliferation via the Notch signaling pathway [295]. *Zhang *et al*.* conducted a study on LINC00261, which revealed its antitumor function through the Notch signaling pathway and its downregulation in HCC [296].

### NF-κB signaling pathway

**Fig. 8:**
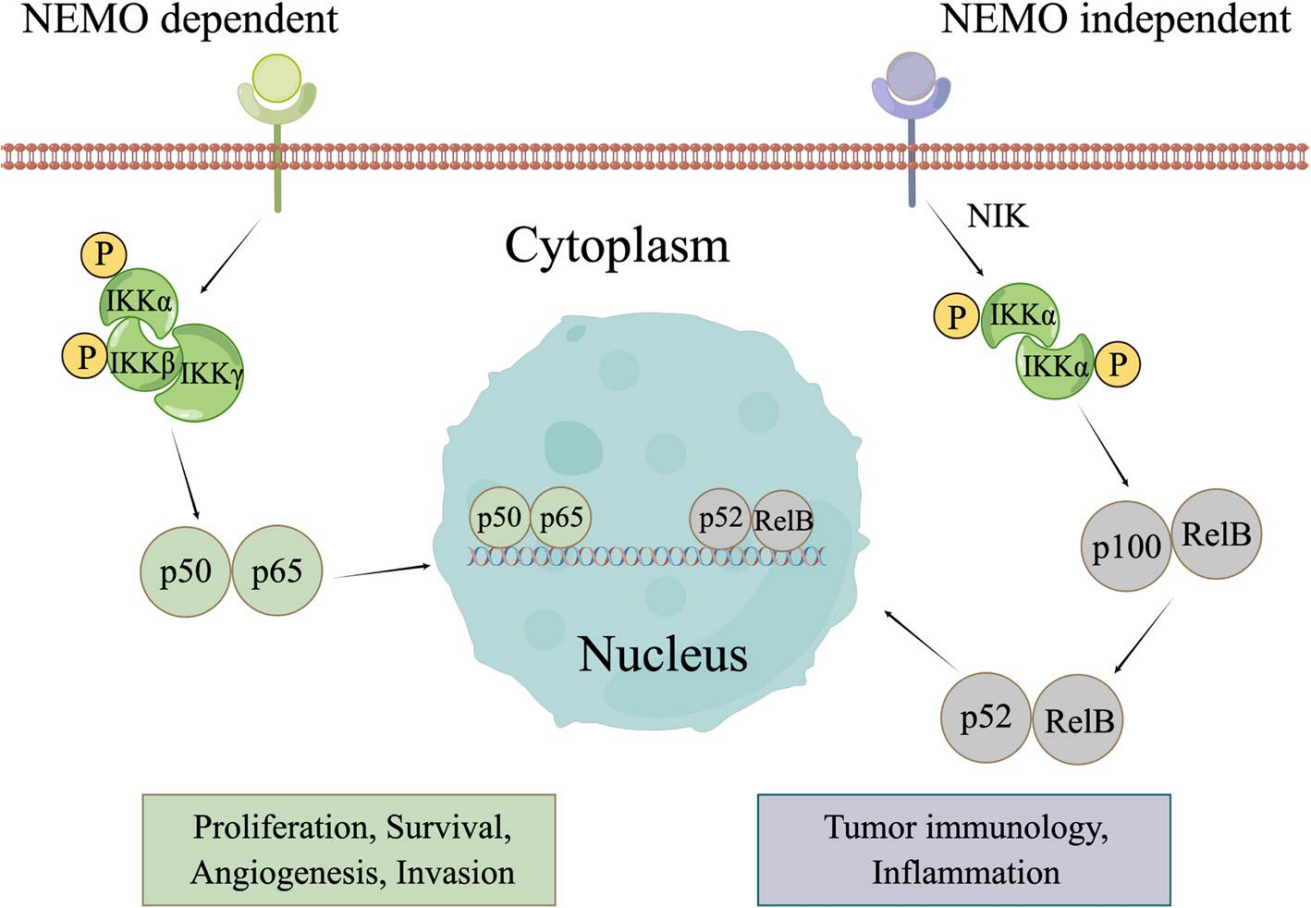
NF-κB signaling pathway. In the NEMO dependent pathway, ligand presence triggers the phosphorylation of IKKα and IKKβ within the IKK complex, leading to the activation and nuclear translocation of p50-p65 dimers to govern the transcription of target genes. Conversely, in the NEMO independent pathway, NIK is activated, which then phosphorylates IKKα-IKKα homodimers. This phosphorylation prompts the formation of p100-RelB dimers, subsequently replaced by p52-RelB dimers that migrate into the nucleus to regulate gene transcription. By figdraw

NF-κB signaling is implicated in numerous inflammatory disorders [297]. Specifically, chronic infection and inflammation involving NF-κB have been linked to a heightened risk of developing certain cancers such as HCC [298]. NF-κB transcription factors regulate inflammatory responses, innate and specific immunity, cell differentiation, survival, and proliferation [299–302]. The NF-κB system is strictly regulated, but its dysregulation has been associated with various diseases, including immune dysregulation, inflammation confusion, and cancerous diseases [303]. In mammals, NF-κB family members (p65/RelA, RelB, cRel, p100/p52, and p105/p50) form homodimers or heterodimers to bind to DNA [304]. The NF-κB signaling pathway is divided into canonical or NF-κB essential modulator (NEMO)-dependent and non-canonical or NEMO-independent pathways. In the canonical pathway, the IKK complex comprises the catalytic subunits IKKα, IKKβ, and IKKγ (also known as NEMO), which activate the canonical signaling. The phosphorylation of IKKα and IKKβ within the IKK complex facilitates the nuclear import of free NF-κB dimers (primarily p50-p65 dimers), initiating the transcription of specific genes. In the non-canonical pathway, activation of NF-κB-inducing kinase (NIK) induces post-translational modification of p100 into the p52 subunit, followed by dimerization with RelB after the formation and phosphorylation of IKKα-IKKα homodimers. The resulting complex then translocates to the nucleus to induce transcription of target genes (Fig. 8) [305]. The role of NF-κB signaling in carcinogenesis is closely associated with inflammation and tumor immunology. Activation of canonical NF-κB signaling has been shown to promote tumor cell survival, proliferation, angiogenesis, and invasion [298, 306]. However, non-canonical NF-κB signaling has been shown to promote certain malignancies; it has been recognized to have an anti-tumor effect through the tumor microenvironment [307–309].

Since HCC commonly arises from chronic hepatitis or cirrhosis [310], aberrant NF-κB signaling pathway activation is necessary for its development [217]. The NF-κB signaling pathway promotes HCC carcinogenesis. Angiogenesis is crucial for tumorigenesis and the development of HCC. *Han *et al*.* found that HCC samples with high micro-vessel density (MVD) had high levels of pro-angiogenic factor RNA-binding motif 23 (RBM23), which was positively correlated with MVD in HCC samples. RBM23 activated the NF-κB signaling pathway by influencing p65/RelA in vitro and in vivo, mediating the expression of pro-angiogenic genes [311]. Recently, *Han *et al*.* further confirmed the potential angiogenic effect of RBM4 in HCC. RBM4 stabilizes RelA/p65 mRNA, activates the NF-κB signaling pathway, and upregulates proangiogenic VEGFA expression [312]. Several compounds that target this pathway have been verified, as outlined in the subsequent section.

## Targeted therapies-current studies and applications

Based on the pathways involved in the carcinogenesis of liver cancer, considerable research has been conducted on targeted inhibitors. Despite this, to date, only tyrosine kinase inhibitors have been adopted for clinical use. Before showing TKIs in detail, we first examine the research status of inhibitors targeting other pathways. We find that although a variety of inhibitors have entered clinical trials, there are relatively few trials specifically aimed at liver cancer. Even in clinical trials targeting solid tumors, those participants who show positive outcomes might be patients with other types of solid tumors. Nonetheless, we maintain that such clinical research is immensely valuable for the clinical translation of liver cancer treatments in the near future. Here, we provide information on clinical studies of representative inhibitors for each pathway, with Table 1 offering a concise summary.

**Table 1:** Targeted molecular inhibitors for specific signaling pathways and their related clinical trials

Signaling pathway	Molecular inhibitor	Target	Disease	Phase of clinical trial	NCT number
Ras/Raf/MEK/ERK signaling pathway	Sotorasib	*KRAS* G12C mutant protein	Solid tumors	Phase 1/2 ACTIVE, NOT RECRUITING	NCT03600883
			Colorectal cancer	+ Panitumumab Phase 1/2 RECRUITING	NCT04185883
	Dabrafenib	BRAF	Glioma	+ Trametinib Phase 2 COMPLETED	NCT02684058
			Melanoma	Phase 3 COMPLETED Phase 3 COMPLETED	NCT01584648 NCT01597908
	Cobimetinib	MEK1/2	Myeloma	+ Venetoclax with or without Atezolizumab Phase 1/2 COMPLETED	NCT03312530
	Ulixertinib	ERK1/2	Solid tumors	Phase 1/2 COMPLETED	NCT01781429
			Pancreatic adenocarcinoma	+ Gemcitabine and Nab-paclitaxel Phase 1 TERMINATED	NCT02608229
	MK-8353	ERK1/2	Solid tumors	+ Selumetinib Phase 1 COMPLETED	NCT03745989
PI3K/AKT/mTOR signaling pathway	Duvelisib	PI3K	Solid tumors	+ SG001 Phase 1/2 NOT YET RECRUITING	NCT05508659
	Roginolisib	PI3K	Metastatic cancers	+ Pemetrexed/ Cisplatin Phase 1 ACTIVE, NOT RECRUITING	NCT04328844
	Dactolisib	PI3K/mTOR	Solid tumors	Phase 1 COMPLETED	NCT00620594
	Buparlisib	PI3K	Solid tumors	+ Everolimus Phase 1 COMPLETED	NCT01470209
	Paxalisib	PI3K	Solid tumors	Phase 2 RECRUITING	NCT03994796
			Glioblastoma	+ Metformin Phase 2 RECRUITING	NCT05183204
	Temsirolimus	mTOR	Liver, Ovarian, Endometrial, Carcinoid, Islet Cell cancer	+ Bevacizumab Phase 2 COMPLETED	NCT01010126
	Sirolimus	mTOR	HCC	Phase 3 COMPLETED	NCT00355862
	AZD2014	mTOR	Gynecological tumors	Phase 1/2 ACTIVE, NOT RECRUITING	NCT02208375
	Capivasertib	AKT	Breast cancer	+ Fulvestrant Phase 3 ACTIVE, NOT RECRUITING	NCT04305496
	MK-2206	AKT	Biliary cancer	Phase 2 COMPLETED	NCT01425879
			Breast cancer	Phase 2 COMPLETED	NCT01277757
	Capivasertib	AKT	Solid tumors, Lymphomas, Multiple myeloma	+ GSK2636771/ Ipatasertib/ Sapanisertib Phase 2 ACTIVE, NOT RECRUITING	NCT02465060
Wnt-β-catenin signaling pathway	LGK974	PORCN	Malignancies dependent on Wnt Ligands	+ PDR001 Phase 1 ACTIVE, NOT RECRUITING	NCT01351103
	ETC-1922159	PORCN	Solid tumors	+ Pembrolizumab Phase 1 ACTIVE, NOT RECRUITING	NCT02521844
	DKN-01	DKK1	Biliary tract cancer	+ Nivolumab Phase 2 RECRUITING	NCT04057365
	OMP-54F28	FZD8	HCC	+ Sorafenib Phase 1 COMPLETED	NCT02069145
			Pancreatic adenocarcinoma	+ Nab-paclitaxel/ Gemcitabine Phase 1 COMPLETED	NCT02050178
	OMP-18R5	FZD	Breast cancer	+ Paclitaxel Phase 1 COMPLETED	NCT01973309
	PRI-724	β-catenin /CBP	Liver cirrhosis	Phase 1/2 COMPLETED	NCT03620474
JAK/STAT signaling pathway	Upadacitinib	JAK1	Active ulcerative colitis	Phase 2/3 COMPLETED	NCT02819635
	Pacritinib	JAK2	Myelofibrosis	Phase 3 RECRUITING	NCT03165734
	Ruxolitinib	JAK1/2	Chronic graft-versus-host disease	Phase 3 COMPLETED	NCT03112603
			Leukemia	Phase 2 COMPLETED	NCT02092324
	Tofacitinib	JAK1/2/3	Ankylosing spondylitis	Phase 3 COMPLETED	NCT03502616
	Napabucasin	STAT3	HCC	Phase 1 COMPLETED	NCT02358395
Hedgehog signaling pathway	Vismodegib	SMO	Solid tumors	Phase 1 COMPLETED	NCT01546519
	Sonidegib	SMO	HCC	+ Sorafenib/ Irinotecan Phase 1 NOT YET RECRUITING	NCT05669339
	IPI-926	SMO	Pancreatic adenocarcinoma	+ FOLFIRINOX Phase 1 COMPLETED	NCT01383538
Hippo signaling pathway	Verteporfin	YAP/TEAD	Glioblastoma	Phase 1/2 RECRUITING	NCT04590664
	VT3989	TEAD	Solid tumors	Phase 1 RECRUITING	NCT04665206
	IK-930	TEAD	Solid tumors	Phase 1 RECRUITING	NCT05228015
Notch signaling pathway	MK-0752	GSI	Pancreatic ductal adenocarcinoma	+ Gemcitabine Phase 1 COMPLETED	NCT01098344
	LY3039478	GSI	Advanced cancer	Phase 1 COMPLETED	NCT01695005
			Solid tumors	+ Taladegib or + LY3023414 or + Abemaciclib Phase 1 COMPLETED	NCT02784795
	REGN421	DLL4	Solid tumors	Phase 1 COMPLETED	NCT00871559
	Demcizumab	DLL4	Solid tumors	Phase 1 COMPLETED	NCT00744562
			Ovarian cancer	+ Paclitaxel Phase 1 TERMINATED	NCT01952249
	OMP-5948	Notch2/3	Solid tumors	Phase 1 COMPLETED	NCT01277146
NF-κB signaling pathway	Andrographolide	Ikks	Knee osteoarthritis	Not Applicable RECRUITING	NCT04833946
	Curcumin	IκBα	Colorectal cancer	+ FOLFOX Phase 1/2 COMPLETED	NCT01490996
	Carfilzomib	Proteasome	Multiple myeloma	+ Dexamethasone/ Isatuximab Phase 3 ACTIVE, NOT RECRUITING	NCT03275285
			Renal cell carcinoma	Phase 2 COMPLETED	NCT01775930
	Aspirin	COX-2	Solid tumors	Phase 3 RECRUITING	NCT02804815
Growth factor receptor-related signaling pathways	Sorafenib	PDGFR-β, VEGFR2, c-KIT	HCC	Phase 3 COMPLETED	NCT00105443
	Lenvatinib	PDGFR-α, c-KIT,RET, VEGFR1-3,FGFR1-4,	HCC	Phase 3 COMPLETED	NCT01761266
			HCC	+ Cadonilimab Phase 1/2 RECRUITING	NCT04444167
	Bevacizumab	VEGFA	HCC	+ Atezolizumab Phase 3 ACTIVE, NOT RECRUITING	NCT04102098
			NSCLC	+ UCPVax Phase 1/2 ACTIVE, NOT RECRUITING	NCT02818426
	Donafenib	VEGFR, PDGFR, Raf kinases	HCC	Phase 2/3 COMPLETED	NCT02645981
	Regorafenib	VEGFR1-3, Raf, c-KIT, PDGFR-β, FGFR, RET, TIE2	HCC	Phase 3 COMPLETED	NCT01774344
			Solid tumors	+ Cetuximab Phase 1 COMPLETED	NCT01973868
	Cabozantinib	c-MET, VEGFR2, AXL, c-KIT	HCC	Phase 3 COMPLETED	NCT01908426
	Ramucirumab	VEGFR2	HCC	Phase 3 COMPLETED	NCT02435433
	Apatinib	VEGFR2	HCC	Phase 3 COMPLETED	NCT02329860
			HCC	+ Camrelizumab Phase 2 UNKNOWN STATUS	NCT04297202
	Erdafitinib	FGFR1-4	Solid tumors	Phase 2 ACTIVE, NOT RECRUITING	NCT04083976
	Capmatinib	c-MET	NSCLC	Phase 2 COMPLETED	NCT02750215
	Erlotinib	EGFR	Chronic HCV infection	Phase 1/2 UNKNOWN STATUS	NCT01835938
	Galunisertib	TGF-βRI	Solid tumors	+ Nivolumab Phase 1/2 COMPLETED	NCT02423343

### Advancements in non-TKI blockers across clinical and preclinical studies

#### Targeted therapy for Ras/Raf/MEK/ERK pathway

Inhibitors targeting RAS, RAF, MEK, and ERK have all been developed. In the past, RAS was deemed challenging to target, but recent studies have shifted this perception, marking a step forward in this area. Recent advancements are highlighted by sotorasib, a small molecule inhibitor specifically targeting the *KRAS* G12C mutant protein, which is prevalent in non-small cell lung cancer (NSCLC) [313], pancreatic cancer [314], and colorectal cancer [315]. This breakthrough has led to numerous clinical studies. A significant study involving patients with *KRAS* G12C-mutant NSCLC showed promising efficacy for the majority of participants (NCT03600883) [316]. Meanwhile, a phase 1 study was launched to test the efficacy of combining sotorasib with the EGFR inhibitor panitumumab in treating chemotherapy-resistant colorectal cancer, which confirmed its safety and efficacy (NCT04185883) [317].

Many clinical investigations on the BRAF inhibitor dabrafenib have utilized its combination with the MEK inhibitor trametinib. A notable study targeting pediatric low-grade gliomas with *BRAF* V600 mutations treated with dabrafenib and trametinib showed enhanced response rates and progression-free survival (PFS) over conventional chemotherapy, maintaining safety (NCT02684058) [318]. In the case of metastatic melanoma patients with *BRAF* V600E or V600K mutations, treatment with dabrafenib and trametinib provided substantial long-term benefits to approximately one-third of the participants, achieving a 5-year OS rate of 71%. This underscores the significant potential of this therapeutic approach (NCT01584648, NCT01597908) [319].

Cobimetinib is known for its role in suppressing MEK1/2 activity [320]. A study examined the safety and effectiveness of cobimetinib on its own, and when combined with venetoclax (a BCL-2 inhibitor), with or without atezolizumab (a PD-L1 inhibitor), in individuals with relapsed or refractory multiple myeloma. Results indicated that cobimetinib as a monotherapy demonstrated a low response rate. However, combinations of cobimetinib-venetoclax and cobimetinib-venetoclax-atezolizumab showed moderate effectiveness. Notably, antitumor effects were noted in patients possessing the t(11;14) chromosomal translocation, indicating its potential as a biomarker for targeted treatments (NCT03312530) [321].

Furthermore, studies on ulixertinib (BVD-523), an ERK inhibitor, have shown its ability to block ERK1/2 functions, reducing the phosphorylation of downstream proteins, which affects cancer cell growth and viability [322]. A phase 1 clinical trial evaluating ulixertinib in patients with advanced solid tumors harboring *MAPK* mutations confirmed its safety profile and antitumor activity (NCT01781429) [323]. Building on this foundation, a new clinical trial has been initiated to evaluate the effectiveness of ulixertinib in conjunction with gemcitabine and nab-paclitaxel (GnP) for the treatment of previously untreated metastatic pancreatic adenocarcinoma. Initial results suggest promising efficacy of adding ulixertinib to GnP, though the occurrence of adverse effects underscores the necessity for additional research on safety and tolerability (NCT02608229) [324]. Additionally, a study involving the ERK 1/2 inhibitor MK-8353 in combination with the MEK inhibitor selumetinib in advanced solid tumors highlighted that safety and tolerability decrease with increased doses. This finding prompts the need for more research to fine-tune dosing and optimize effectiveness (NCT03745989) [325]. In summary, targeting the Ras/Raf/MEK/ERK pathway with specific agents is closely linked to particular genetic aberrations. Ongoing research focuses on specific cancer types, and expanding these studies into broader cancer applications remains a critical area of development.

#### Targeted therapy for PI3K/AKT/mTOR signaling pathway

PI3K, mTOR, and AKT are all key targets of the pathway, with PI3K inhibitors being categorized into isoform-specific PI3K inhibitors, dual PI3K/mTOR inhibitors, and pan-PI3K inhibitors [326]. Idelalisib and duvelisib are isoform-specific PI3K inhibitors while idelalisib has shown promising pro-apoptotic effects in HCC [327]. Although idelalisib and duvelisib have been extensively researched in hematological malignancies [328, 329], their investigation in solid tumors remains limited. A recent initiative aims to evaluate the efficacy of duvelisib combined with the PD-1 inhibitor SG001 in treating advanced solid tumors (NCT05508659). Additionally, roginolisib (IOA-244), another isoform-specific PI3K inhibitor, has displayed the capability to trigger apoptosis in mesothelioma cells and counteract the immunosuppressive microenvironment in vitro [330]. An upcoming clinical study is going to assess IOA-244's performance alone and in combination with pemetrexed/cisplatin in advanced cancer patients (NCT04328844). Dactolisib (NVP-BEZ235), a dual PI3K/mTOR inhibitor, when paired with an anti-IL-6 antibody, has exhibited potent cytotoxic effects against HCC cells [331]. The safety and efficacy of its oral administration in patients with solid tumors, including advanced breast cancer, are currently under phase 1 investigation (NCT00620594). Buparlisib (BKM120) and paxalisib (GDC-0084) are pan-PI3K inhibitors. Preliminary studies have confirmed BKM120's potent antitumor effect on HCC [332]. A recent phase 1 clinical study on the combination of buparlisib (BKM120) and everolimus, a first-generation mTOR inhibitor, for the treatment of advanced solid tumors (NCT01470209) has shown promising results. The findings indicate safety and tolerability among patients, warranting further research into this therapeutic approach [333]. Additionally, clinical trials related to paxalisib are currently underway or in the process of recruiting participants. For instance, a study investigating its efficacy in patients with solid tumors that have metastasized to the brain is actively recruiting (NCT03994796). Furthermore, an ongoing phase 2 clinical trial is evaluating the effectiveness of paxalisib in treating patients newly diagnosed with glioblastoma (NCT05183204).

The initial generation of mTOR inhibitors, mainly consisting of rapamycin and its derivatives, predominantly focuses on mTORC1, which is essential for cellular growth [334]. A clinical study explored the efficacy of temsirolimus combined with the anti-angiogenic agent bevacizumab in treating certain type of cancer (including those of liver), reporting an objective response rate (ORR) of 19% and a median OS of 14 months (NCT01010126) [335]. Concerning sirolimus, the prolonged use of this medication for a period exceeding three months significantly improves the outcomes of individuals undergoing liver transplantation for HCC, notably among those exhibiting greater tumor activity as evidenced by elevated alpha-fetoprotein (AFP) levels (NCT00355862) [336]. Unlike the first generation, the second generation of mTOR inhibitors, such as AZD2014, an ATP-competitive mTOR kinase inhibitor, simultaneously blocks both mTORC1 and mTORC2 complexes, inhibiting the feedback activation of the PI3K/AKT signaling pathway and thus offering a more potent inhibition than the first generation [337]. Mouse models have confirmed AZD2014's potential therapeutic effect on HCC [338], and studies have also shown its anticancer activity in ovarian cancer [339]. A current clinical study is investigating the adverse reactions and optimal dosage of AZD2014 or the oral AKT inhibitor AZD5363 in recurrent gynecological tumors (NCT02208375) [340]. Building upon previous generations, third-generation mTOR inhibitors, such as rapalinks, are designed to simultaneously block the ATP site, forming a unique conformation to achieve localized high concentration [341]. However, the effectiveness of rapalinks in solid tumors requires further investigation.

A phase 3 clinical trial focused on patients with advanced breast cancer characterized by positive hormone receptors and negative for human epidermal growth factor receptor 2 (HER2), who had relapsed or progressed during or following treatment with aromatase inhibitors. This investigation explored the efficacy and safety of an oral AKT inhibitor, capivasertib, in combination with the estrogen receptor (ER) antagonist, fulvestrant, as a treatment regimen. The results indicated that, in the overall population, the capivasertib-fulvestrant combination yielded a median progression-free survival (PFS) of 7.2 months, in contrast to 3.6 months for those receiving placebo-fulvestrant. Among individuals with alterations in the AKT pathway, the median PFS in the capivasertib-fulvestrant group increased to 7.3 months, compared to 3.1 months in the placebo-fulvestrant group. Despite capivasertib increasing the risk of certain adverse events, these were manageable. Overall, the potential value of AKT inhibitors in enhancing treatment strategies for hormone receptor-positive advanced breast cancer is significant (NCT04305496) [342]. The clinical research on the AKT inhibitor MK-2206 for cancer treatment has encountered challenges. It did not demonstrate significant efficacy in patients with unresectable, advanced refractory biliary cancer (NCT01425879) [343]. Additionally, another clinical trial investigating MK-2206 in patients with advanced breast cancer, specifically those with *PIK3CA* or *AKT* mutations, and/or *PTEN* mutations or loss, showed a minimal antitumor response (NCT01277757) [344]. Moreover, a phase 2 clinical trial evaluated the effectiveness of combined treatment involving GSK2636771 (a PI3Kβ inhibitor), capivasertib (an AKT inhibitor), ipatasertib (an AKT inhibitor), and sapanisertib (an mTORC1/2 inhibitor) [175]. This clinical trial aims to explore the healing impacts of utilizing these four inhibitors in patients with solid tumors, lymphomas, or multiple myeloma, and is currently underway (NCT02465060) [345]. It appears that employing combination therapies, rather than monotherapy, possesses the capability to overcome the significant obstacles linked to HCC therapy.

#### Targeted therapy for the Wnt-β-catenin signaling pathway

PORCN is an O-acyltransferase essential for the secretion of Wnt ligands [346]. Clinical studies on PORCN inhibitors are currently underway. A clinical trial aims to identify the recommended dose of the PORCN inhibitor LGK974, both as a single agent and in combination with PDR001 (a PD-1 antibody), in patients with malignancies that rely on Wnt ligands. This clinical trial is actively underway (NCT01351103) [347]. In another study, the PORCN inhibitor ETC-1922159 has demonstrated potential in causing tumor necrosis by disrupting tumor angiogenesis [348]. A clinical trial involving ETC-1922159 selected patients with advanced solid tumors and the assessment of the safety and tolerability of ETC-1922159 is ongoing (NCT02521844).

DKK1 has been reported to promote cancer progression by enhancing β-catenin expression [349]. A clinical study is evaluating the combined efficacy of the PD-1 inhibitor nivolumab and the DKK1 inhibitor DKN-01 in patients with advanced biliary tract cancer. Participants are now under recruiting (NCT04057365) [350]. OMP-54F28 is a FZD8 inhibitor [351]. A phase 1 study exploring the combination of OMP-54F28 with sorafenib for treating HCC did not demonstrate positive results (NCT02069145). Another clinical study showed that patients with stage IV pancreatic cancer tolerated the combination of OMP-54F28 with nab-paclitaxel and gemcitabine well, potentially offering a new treatment for metastatic pancreatic adenocarcinoma (NCT02050178) [352]. Another FZD inhibitor, OMP-18R5, in a phase 1 trial regarding its combination with paclitaxel in patients with locally recurrent or metastatic HER2-negative breast cancer, showed good tolerance in patients. Nonetheless, a high incidence of fractures might limit its application (NCT01973309) [353]. PRI-724, by disrupting the interaction between β-catenin and CBP, inhibits the Wnt-β-catenin signaling pathway [354]. A clinical study involving patients with hepatitis C or B virus-derived liver cirrhosis showed good tolerance to a 12-week intravenous administration of 280 mg/m^2^/4 h PRI-724. However, the anti-fibrotic effects of PRI-724 require further evaluation (NCT03620474) [355].

#### Targeted therapy for the JAK/STAT signaling pathway

Current research on JAK inhibitors is extensive, broadly categorizing them into selective and non-selective classes. The selective JAK1 inhibitor, upadacitinib, has demonstrated significant efficacy and safety in phase 3 clinical trials for treating moderate to severe ulcerative colitis (NCT02819635, NCT03653026) [356], with its clinical study results in Crohn's disease also being highly encouraging (NCT03345849, NCT03345836, NCT03345823) [357]. The JAK2 inhibitor, pacritinib, has shown promising application prospects in patients with advanced myelofibrosis who are resistant or intolerant to ruxolitinib (NCT04884191) [358], and is currently recruiting for a phase 3 trial involving patients with myelofibrosis and severe thrombocytopenia (NCT03165734). While the clinical efficacy is unclear in HCC patients, recent research has revealed that pacritinib antagonizes liver fibrosis in animal models, suggesting a potential inhibitory effect on the progression of HCC [359].

Ruxolitinib and tofacitinib are non-selective JAK inhibitors, each with its own specific targets and efficacy profiles. Ruxolitinib primarily inhibits JAK1 and JAK2. It has demonstrated excellent efficacy in treating glucocorticoid-refractory chronic graft-versus-host disease (NCT03112603) [360]. Additionally, it has shown good tolerability in a phase 2 study for the treatment of chronic neutrophilic leukemia and atypical chronic myeloid leukemia. Intriguingly, it appears that patients with chronic neutrophilic leukemia, with or without the *CSF3R*-T618I mutation, exhibit a better response (NCT02092324) [361]. Furthermore, *Wilson *et al*.* initially demonstrated the capability of ruxolitinib to inhibit HCC cell colony formation, highlighting its potential in HCC treatment [362]. Tofacitinib, which targets JAK1, JAK2, and JAK3, has shown promising results in treating autoimmune diseases such as ankylosing spondylitis (NCT03502616) [363] and psoriatic arthritis (NCT01877668, NCT01882439) [364]. However, it is crucial to note that clinical studies have raised concerns regarding its safety profile. Compared to treatments with tumor necrosis factor inhibitors (TNFi) for rheumatoid arthritis, tofacitinib application is linked to a higher likelihood of malignant tumor formation [365], emphasizing the need for careful consideration and monitoring in its clinical use.

Napabucasin (BBI608) is a STAT3 inhibitor [366]. A clinical study enrolled adult patients in Japan with advanced HCC to evaluate the efficacy of BBI608 in combination with sorafenib. The study results showed its safety and potential role in treating advanced, unresectable HCC (NCT02358395) [367]. The clinical study results for STAT3 inhibitors OPB-111077 (NCT01942083) [368] and OPB-31121 (NCT01406574) [369] in HCC were not optimistic, with unclear therapeutic effects. Overall, it seems that JAK-related inhibitors are mainly used for treating autoimmune diseases at present, and research on JAK/STAT pathway inhibitors in solid tumors is still relatively scarce.

#### Targeted therapy for the Hedgehog signaling pathway

Currently, targeted therapies for the Hedgehog pathway predominantly focus on inhibiting SMO, with drugs such as vismodegib, sonidegib, and IPI-926. Vismodegib is notably the first drug approved for the treatment of locally advanced basal cell carcinoma (laBCC) [370]. Despite phase 1 clinical trials assessing vismodegib in patients with advanced solid tumors, including HCC, with various levels of renal and hepatic function, the studies did not reach a satisfactory conclusion, as no participants completed the study due to disease progression, death, or other factors necessitating discontinuation of the treatment (NCT01546519). Therefore, the efficacy of vismodegib against other solid tumors requires further investigation. Sonidegib offers a different targeted treatment approach for basal cell carcinoma management [371]. A current clinical trial is evaluating the effects of a custom combination therapy consisting of sonidegib, sorafenib (a multi-kinase inhibitor) and irinotecan, for the treatment of HCC (NCT05669339). In contrast, the SMO inhibitor IPI-926, used in conjunction with the FOLFIRINOX (5-fluorouracil, leucovorin, irinotecan, oxaliplatin) chemotherapy regimen for advanced pancreatic adenocarcinoma, did not achieve the expected outcomes, raising questions about its future application in this setting (NCT01383538) [372]. Moreover, significant strides have been made in the development of compounds targeting Gli1. Research conducted by *Oladapo *et al*.* has demonstrated the inhibitory effects of HPI-1, GANT61, and JK184 on Gli1 in triple-negative breast cancer cell lines. These discoveries highlight the potential of Gli1 as a target in anticancer strategies and warrant further investigation and development [373].

#### Targeted therapy for the Hippo signaling pathway

Verteporfin, a photodynamic therapy drug used for treating macular degeneration [374], was also shown to disrupt the interaction between YAP and TEAD [375]. In a cholangiocarcinoma mouse model, it demonstrated anti-tumor activity by increasing the ratio of M1 macrophages and CD8 T cells [376]. Furthermore, *Zhang *et al*.* investigated the mechanism of interaction between the pregnane X receptor (PXR) and YAP in liver regeneration. It was found that verteporfin significantly affected PXR-induced liver hypertrophy and liver regeneration after 70% partial hepatectomy (PHx) [377], suggesting verteporfin's potential future use in treating HCC. In addition, clinical trials for verteporfin in the treatment of recurrent high-grade *EGFR*-mutated glioblastoma are currently recruiting participants (NCT04590664). A new drug VT3989 works by inhibiting TEAD palmitoylation, which in turn blocks YAP function. Given the close relationship between *NF2* mutations and YAP activity, which leads to tumor growth [378], a clinical study explored the efficacy of VT3989 in individuals with advanced solid tumors with a high prevalence of malignant mesothelioma and other tumors with *NF2* mutations, showing good tolerance and sustained anti-tumor responses (NCT04665206) [379].

Lastly, IK-930 is an oral inhibitor of TEAD, and a clinical study on its efficacy in patients with advanced solid tumors is currently open for recruitment (NCT05228015). Research is underway on other kind of Hippo signaling inhibitors. Recently, *Chen *et al*.* found a suppressive effect of α-hederin on the Hippo signaling pathway. α-hederin treatment effectively reduced nuclear YAP levels through MST1/2 phosphorylation, inducing the inhibition of proliferation and apoptosis of HCC cells in vitro. Additionally, the tumor volume and mass were notably decreased in a xenograft mouse model [380].

#### Targeted therapy for the Notch signaling pathway

The first class of Notch pathway inhibitors includes γ-secretase inhibitors (GSIs) such as MK-0752 and LY3039478, which block a key enzyme in the Notch signaling pathway. A phase 1 clinical study investigated the combination of MK-0752 with gemcitabine in treating patients with pancreatic ductal adenocarcinoma. The results suggested that this therapy is relatively safe and effective, offering a potential new treatment option for those patients (NCT01098344) [381]. Previous studies have shown that LY3039478 possesses a strong inhibitory effect on the Notch pathway and is well tolerated in advanced or metastatic tumors (NCT01695005) [382]. However, clinical studies combining LY3039478 with taladegib (a SMO inhibitor), LY3023414 (a dual PI3K/mTOR inhibitor), or abemaciclib (a selective inhibitor of cyclin-dependent kinases 4 and 6) in the treatment of solid tumors in late stage have shown a high incidence of adverse reactions. Participants showed poor tolerance and the combination treatments were clinically ineffective (NCT02784795) [383].

Inhibitors targeting the ligands or receptors of the Notch pathway represent another therapeutic approach. REGN421 and demcizumab (OMP-21M18) function as DLL4 inhibitors. Findings from a clinical study on the safe administration amount of REGN421 as a sole treatment in advanced solid tumor patients revealed, despite encountering some serious adverse events, observable partial tumor responses and multiple instances of disease stabilization (NCT00871559) [384]. Previously, demcizumab has shown promise in halting the progression of solid tumors in a clinical trial (NCT00744562) [385]. More recently, a study assessed the maximum tolerated dose (MTD) and maximum administered dose (MAD) of demcizumab combined with paclitaxel in treating platinum-resistant ovarian cancer, demonstrating notable antitumor efficacy [386]. Another clinical trial explored the tolerable dosage range of OMP-5948, a novel antibody that selectively inhibits Notch2 and Notch3, in the treatment of solid tumors, confirming its suppressive effect on the Notch pathway (NCT01277146) [387]. Other inhibitors targeting the Notch pathway are currently under investigation. *Zhang *et al*.* reported an inhibitor of the Notch activating/cleaving enzyme ADAM-17, named ZLDI-8 which inhibits S2 cleavage. It lowers the levels of proteins associated with pro-survival/anti-apoptosis and EMT, while augmenting sorafenib's efficacy in vivo [388].

#### Targeted therapy for the NF-κB signaling pathway

Andrographolide acts as an inhibitor of Ikks. *Chowdhury *et al*.* demonstrated that treatment with andrographolide notably reduces the nuclear localization of NF-κB by modulating the PKA/PP2A/IKK axis in a cisplatin-resistant human HCC cell line (HepG2CR) [389]. Although there's extensive clinical research indicating andrographolide's positive effects in conditions like multiple sclerosis (NCT02273635) [390] and knee osteoarthritis (NCT04833946) [391], studies in solid tumors are limited. The activators of IκBα, which serves as a negative regulator of NF-κB, can also inhibit the NF-κB pathway. Research indicates that curcumin, derived from turmeric, inhibits NF-κB signaling in ulcerative colitis by enhancing IκBα [392]. A phase 2 clinical trial combining curcumin with FOLFOX chemotherapy in treating metastatic colorectal cancer showed a notable rise in median OS and a marginal increase in PFS (NCT01490996) [393].

Proteasome is also the target of NF-κB signaling. Carfilzomib, a proteasome inhibitor, effectively prevents degradation of phosphorylated IκB, thus blocking NF-κB's entry into the nucleus [394]. Demonstrating its efficacy in multiple myeloma, a clinical study combining carfilzomib-dexamethasone with the anti-CD38 antibody isatuximab has shown notable improvements in PFS, complete response rates, and minimal residual disease (MRD) negativity over carfilzomib-dexamethasone alone (NCT03275285) [395]. Despite this success, carfilzomib's application in clear-cell renal cell carcinoma yielded unfavorable results, with disease advancement in every patient (NCT01775930) [396].

Interestingly, aspirin can suppress cyclooxygenase-2 (COX-2), an effector molecule produced following the activation of the NF-κB pathway, thus indirectly inhibit the NF-κB pathway, specifically curtailing LPS-induced macrophage activation in the early stage of inflammation [397]. There is a clinical trial in studying aspirin's potential to prevent recurrence and extend survival in solid tumors (NCT02804815). Additionally, natural compounds oleanolic acid (OA) and ursolic acid (UA) have been shown to target NF-κB's p65 subunit [398]. *Fontana *et al*.* demonstrated that certain derivatives of OA and UA blocked HCC cell growth by inhibiting NF-κB activation [399]. OA has also been explored for insulin resistance and type 2 diabetes treatment (NCT06030544). However, the antitumor properties of OA and UA require more clinical studies to support.

### Advancements of TKIs for HCC management

Currently, approved drugs for the clinical use of targeted therapy for HCC are divided into first- and second-line drugs. The first-line targeted agents mainly include sorafenib, lenvatinib, bevacizumab, atezolizumab and donafenib, whereas the second-line targeted agents mainly include regorafenib, cabozantinib, ramucirumab and apatinib. In fact, these TKIs function by blocking growth factor receptors, consequently inhibiting downstream pathways such as the PI3K/AKT/mTOR and Ras/Raf/MEK/ERK pathway as well [400].

#### First-line targeted therapies

Sorafenib targets PDGFR-β, VEGFR2, and c-KIT [401]. Evidence has confirmed that sorafenib acts on the Ras/Raf/MEK/ERK and JAK/STAT3 pathways through tyrosine kinase receptors, inhibiting angiogenesis, cell growth, and metastasis of HCC [402, 403], successfully extending the overall median survival in patients with advanced HCC [404]. Sorafenib was approved as the first systemic drug for the treatment of advanced unresectable HCC in 2007 [404]. The Sorafenib Hepatocellular Carcinoma Assessment Randomized Protocol (SHARP) trial, assessing sorafenib in HCC, showed a roughly 3-month increase in median survival and time to radiologic progression versus placebo (NCT00105443) [404]. Due to resistance issues that arise during the application of sorafenib, recent clinical studies on treatment regimens for advanced HCC have identified several other targeted therapies and combination therapies that have shown superior efficacy to sorafenib.

Lenvatinib is a selective, multi-target tyrosine kinase inhibitor of PDGFR-α, c-KIT, VEGFR1-3, FGFR1-4, and Rearranged During Transfection (RET) [405]. Lenvatinib was approved by the FDA as a first-line treatment for unresectable HCC in 2018 (NCT01761266) [406]. Lenvatinib inhibits tumor angiogenesis and tumor cell proliferation, which may induce its anti-tumor effects [407]. Collectively, these substances curtailed the invasion and spread of human HCC cells [408]. The REFLECT trial showed promising outcomes in patients with unresectable HCC. Lenvatinib outperformed sorafenib in terms of PFS, TTP (time-to-progression), and ORR [409]. This study implied that lenvatinib exhibits a markedly superior antitumor capability compared to sorafenib. Recently, a phase 1b/2 single-arm clinical trial investigated the effectiveness and tolerability of cadonilimab (an inhibitor targeting both PD-1 and CTLA-4 checkpoints) [410] in conjunction with lenvatinib in treating advanced hepatocellular carcinoma (aHCC). Subjects were allocated into two groups: group A received cadonilimab at 6 mg/kg bi-weekly, while group B was administered 15 mg/kg tri-weekly, each alongside lenvatinib. The observed response rates were 35.5% in group A and 35.7% in group B, respectively. Median PFS reached 8.6 months in group A and 9.8 months in group B, with the median duration of response (DoR) noted at 13.6 months for group A and 13.67 months for group B. Significant treatment-related adverse events (TRAEs) of grade 3 or above were noted in 66.1% of participants, with 39.0% experiencing severe TRAEs. In summary, this innovative combined treatment approach showed notable efficacy with controlled toxicity, positioning it as a viable first-line option for aHCC (NCT04444167) [411].

Bevacizumab, a humanized monoclonal antibody that attaches to all forms of circulating, soluble VEGFA, thus effectively blocks the VEGF pathway and its angiogenic role in tumors [412]. A systematic review focusing on low-grade serous ovarian cancer (LGSOC) treated with bevacizumab encompassed 153 patients, revealing an overall median response rate of 47.5%, surpassing traditional chemotherapy approaches [413]. Clinical studies on HCC often combine bevacizumab with the PD-L1 inhibitor atezolizumab [414]. A phase 3 trial evaluated the effectiveness of this regimen versus active monitoring in HCC patients with a high recurrence risk after radical resection or ablation. The IMbrave050 study (NCT04102098) indicated that atezolizumab combined with bevacizumab notably enhanced recurrence-free survival. Despite a higher incidence of grade 3 or 4 adverse events in the treatment group compared to the surveillance group (41% vs. 13%), IMbrave050 is regarded as the first phase 3 trial to show positive adjunctive therapy results in HCC [415]. Atezolizumab plus bevacizumab is now widely recognized as a standard first-line treatment for unresectable HCC [416]. Aiming to further enhance the objective response rate in advanced HCC, a randomized non-comparative phase 2 study (NCT05528952) incorporating the CD4 helper T-inducer cancer anti-telomerase vaccine (UCPVax) is underway [417]. UCPVax, a therapeutic cancer vaccine composed of two distinct peptides from human telomerase reverse transcriptase (TERT), has shown safety and the ability to stimulate an immune reaction in non-small cell lung cancer trials (NCT02818426) [418]. This clinical study seeks to offer new therapies to improve the treatment response rate for unresectable HCC.

Donafenib, recognized as a deuterium-modified form of sorafenib, acts as an oral broad-spectrum multikinase inhibitor targeting several receptor tyrosine kinases, including VEGFR, PDGFR, and Raf kinases [419]. Research juxtaposed the effectiveness and safety of donafenib versus sorafenib for first-line therapy in individuals with inoperable or metastatic hepatocellular carcinoma (NCT02645981) [420]. The median OS for patients receiving donafenib was significantly longer than for those on sorafenib (12.1 months vs. 10.3 months), alongside a lower incidence of drug-related adverse events of grade 3 or higher (38% vs. 50%). Although the differences in median PFS and disease control rate were not statistically significant, the outcomes favored the donafenib treatment group. These results underscore donafenib's promise as an encouraging solitary treatment choice for first-line therapy in advanced HCC patients. In June 2021, donafenib was first sanctioned in China for the treatment of patients with inoperable hepatocellular carcinoma who hadn't received prior systemic therapy [419].

#### Second-line targeted therapies

Regorafenib, another orally administered diphenylurea multi-kinase inhibitor, acts on various receptor tyrosine kinases, including those involved in angiogenesis (VEGFR1-3, tyrosine kinase with immunoglobulin-like and EGF-like domains 2 also known as TIE2), stromal functions (PDGFR-β, FGFR), and oncogenesis (c-KIT, RET, Raf) [421, 422]. In 2016, a randomized, double-blind, placebo-controlled phase 3 study showed statistically significant extensions in OS (10.6 vs. 7.8 months) and PFS (3.1 vs. 1.5 months) (NCT01774344) [423]. Following the results from the RESORCE trial, the FDA in 2017 granted approval to regorafenib for treating patients with advanced hepatocellular carcinoma (HCC) who had experienced progression on sorafenib [423]. In a phase 1b study, the safety and pharmacokinetics (PK) of combining regorafenib with cetuximab (an EGFR inhibitor) were assessed in patients with advanced refractory solid tumors, including gastrointestinal stromal tumors, colorectal cancer, and HCC [424]. A total of 42 patients were treated, with 31 receiving regorafenib intermittently (120 mg, *n* = 8; 160 mg, *n* = 23) and 11 continuously (60 mg, *n* = 5; 100 mg, *n* = 6), alongside weekly cetuximab (250 mg/m^2^). In the intermittent dosing cohort, a 120 mg dosage led to grade 3 hand-foot skin reactions as dose-limiting toxicities (DLTs), while no DLTs were observed at the 160 mg dosage. Therefore, the daily dose of 160 mg regorafenib in combination with cetuximab (250 mg/m^2^) was designated as the MTD. This novel combined treatment strategy of regorafenib and cetuximab may present a new therapeutic option for patients with treatment-resistant solid tumors in advanced stages (NCT01973868) [425].

Cabozantinib, an inhibitor of tyrosine kinase receptors such as c-MET, VEGFR2, AXL, and c-KIT, promotes the growth and progression of HCC and resistance to antiangiogenic therapy [426]. In 2018, a randomized, double-blind, phase 3 study contrasted cabozantinib against placebo in patients with advanced HCC who had received prior treatment. The results revealed that median OS was 10.2 and 8.0 months with cabozantinib and placebo, respectively. Median progression-free survival was 5.2 and 1.9 months with cabozantinib and placebo, respectively (NCT01908426) [427]. Based on these findings, the FDA authorized cabozantinib in 2019 as a second-line treatment option for advanced HCC patients with Child–Pugh A liver function who have been treated with sorafenib yet continued to progress [428].

Ramucirumab is a human IgG1 monoclonal antibody designed to target VEGFR2 [429]. Despite not achieving a notable increase in OS in the REACH trial [430], the results of the REACH-2 trial are encouraging. Ramucirumab improved OS in advanced HCC patients with higher than 400 ng/mL of α-fetoprotein concentrations, who had been treated with sorafenib in the randomized, double-blind phase 3 REACH-2 trial (NCT02435433) [431]. This study represents the initial phase 3 trial to yield positive results within a biomarker-selected cohort of HCC patients, culminating in the authorization of secondary treatment alternatives for advanced HCC.

Apatinib is a highly selective inhibitor of VEGFR2 [432]. *Yang *et al*.* confirmed that the antiproliferative effect of apatinib is concentration-dependent across six liver cancer cell lines (SMMC-7721, Hep3B, SK-Hep-1, Huh-7, HepG2, PLC/PRF/5), and this effect is closely associated with the level of VEGFR2 expression. In vivo experiments also verified apatinib's role in inhibiting HCC growth and angiogenesis [433]. A multi-center, double-blind, randomized, placebo-controlled phase 3 trial investigated the efficacy of apatinib as a secondary therapy for HCC. The OS in the apatinib group showed significant improvement compared to the placebo group (8.7 months vs. 6.7 months), with a hazard ratio of 0.785. Common grade 3 or 4 treatment-related adverse events, including hand-foot syndrome, hypertension and decreased platelet count, were more prevalent in the apatinib group. Overall, apatinib is considered a viable and safe treatment option for patients with advanced HCC (NCT02329860) [434]. A single-arm, open-label, phase 2 clinical trial assessed the safety and efficacy of camrelizumab (a PD-1 monoclonal antibody) combined with apatinib in the perioperative setting for resectable HCC. In this study of 18 HCC patients completing neoadjuvant therapy, 16.7% and 33.3% achieved an ORR according to the standard of Response Evaluation Criteria in Solid Tumors (RECIST) V.1.1 and its modified version, respectively. Among the 17 patients who underwent surgical removal, 17.6% reported major pathological reactions (MPR), and 5.9% achieved a complete pathological response. The one-year relapse-free survival rate was 53.85%. Grade 3 or 4 side effects occurred in 16.7% of participants. Furthermore, RNA sequencing suggested that dendritic cell (DC) infiltration was higher in responding tumors than in non-responding ones, possibly acting as a predictive indicator for the treatment response to the camrelizumab and apatinib combination (NCT04297202) [435].

#### Other targeted therapies for tyrosine kinase receptors

Erdafitinib, a selective ATP-competitive antagonist of FGFR1-4, achieved distinction as the initial FGFR kinase inhibitor to gain FDA approval for treating urothelial carcinoma [436]. A global, single-arm, phase 2 trial explored the safety and efficacy of erdafitinib among individuals with advanced solid tumors (urothelial cancer excluded) presenting FGFR1-4 alterations. Participants received an oral daily dose of erdafitinib (8 mg, with a possibility for a pharmacodynamically informed escalation to 9 mg/day) across a consistent 21-day cycle, continuing until either the disease progressed or unacceptable toxicity was observed. Out of 217 participants, 64 demonstrated an objective response, with a subset encountering adverse reactions linked to the treatment. In essence, erdafitinib demonstrates potential clinical advantage for patients with advanced solid tumors featuring sensitive FGFR alterations, offering a new avenue for those who have no remaining conventional therapy options (NCT04083976) [437].

Capmatinib is a highly selective c-MET inhibitor [438]. The efficacy of capmatinib was evaluated in a phase 2 clinical study across several cohorts of patients with *MET*-dysregulated advanced NSCLC (*MET* exon 14 skipping mutation or *MET* amplification), with administration of the 400 mg oral tablet twice per day. In individuals who had undergone one or two prior lines of therapy, the overall response rate was 41% (69 patients), whereas it was 68% (28 patients) in those without previous treatment, with median response durations being 9.7 months and 12.6 months, respectively. In the patient cohort with lower *MET* amplification (gene copy number below 10), the response rate to treatment was noted in only 7 to 12% of the cases. Conversely, in patients with higher levels of *MET* amplification (gene copy number 10 or above), the response rate improved to 29% for those who had undergone previous treatments and 40% for treatment-naive patients. Adverse events primarily fell into the milder grade 1 or 2 category. These patterns suggest capmatinib's significant role in curbing tumor activity in advanced NSCLC, especially pronounced in patients with *MET* exon 14 skipping mutation who are untreated. It appears that, within the context of *MET*-amplified advanced NSCLC, capmatinib's effectiveness is considerably greater in tumors with higher gene copy numbers (NCT02750215) [439].

A phase 1 trial assessed an EGFR inhibitor erlotinib in chronic hepatitis C (CHC) patients, exploring its safety and antiviral effects. The study involved patients treated with a placebo, erlotinib 50 mg/d, and erlotinib 100 mg/d. Despite the lack of substantial HCV-RNA level reduction during the study, two-thirds of the subjects receiving erlotinib at a daily dose of 100 mg showed a decrease exceeding 0.5 log in HCV-RNA 14 days subsequent to the treatment's conclusion. This suggests erlotinib's potential for safely treating chronic hepatitis C and providing a new approach to preventing HCC development from chronic hepatitis C (NCT01835938) [440].

A phase 1b/2 study evaluated the effectiveness of combining the selective TGF-βRI kinase inhibitor galunisertib with PD-1 inhibitor nivolumab in advanced refractory solid tumors and recurrent/refractory NSCLC. In phase 1, no dose-limiting toxicities were identified. Phase 2 was aimed at NSCLC patients who had previously received platinum-based therapies but were inexperienced with immuno-oncology treatments. Remarkably, 24% of the NSCLC participants experienced a partial response, and 16% maintained stable disease, with the average duration of response being 7.43 months. The study recorded a median progression-free survival of 5.26 months and a median OS of 11.99 months. After treatment, induced interferon gamma response genes and a reduction in cell adhesion gene expression were noted. Despite the limited clinical samples, the results of this study highlight the potential and tolerability of the galunisertib and nivolumab combination in treating some solid tumors (NCT02423343) [441].

## Mechanisms of TKIs resistance in the treatment of HCC

**Fig. 9:**
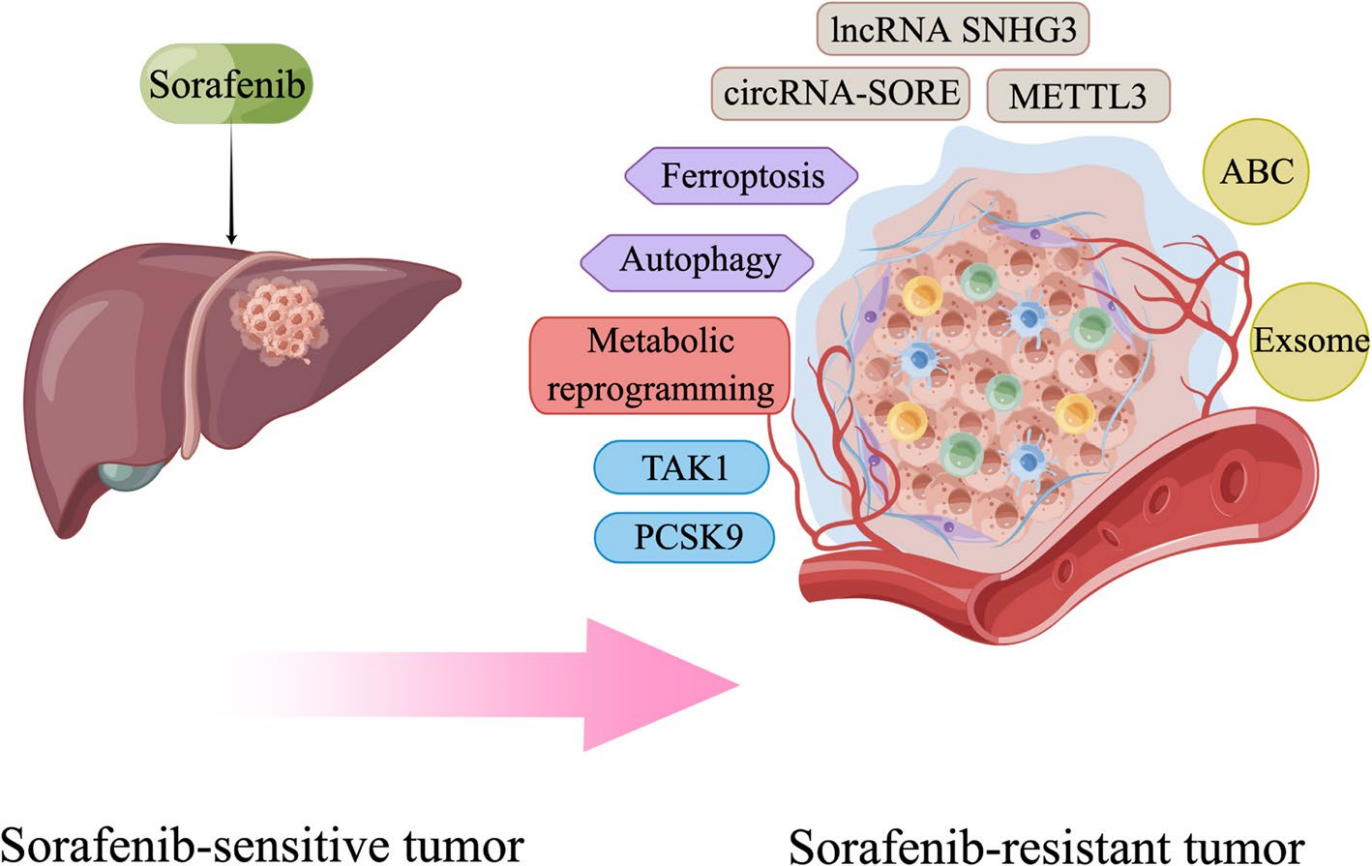
Mechanisms of sorafenib resistance in HCC. Complex mechanisms lie in the resistance to sorafenib in HCC. Not only do they include aspects of epigenetics such as circRNA-SORE, lncRNA SNHG3, and METTL3-mediated processes, but transporters like ABC and exosomes are also involved in resistance. Additionally, from regulated cell death, represented by ferroptosis and autophagy, to tumor metabolic reprogramming, and factors like TAK1 and PCSK9 have also been found to participate in HCC's resistance to sorafenib. By figdraw

Sorafenib, the inaugural FDA-approved targeted therapy, initially demonstrated efficacy against HCC. However, resistance usually typically emerges within six months [442], significantly undermining its therapeutic effectiveness in advanced HCC, thus limiting its clinical utility. A similar resistance phenomenon has been observed with other TKIs, such as lenvatinib [443] and regorafenib [444]. In response, extensive research into TKIs resistance has been undertaken, especially in sorafenib, uncovering multiple underlying mechanisms (Fig. 9). These discoveries aim to pave the way for overcoming resistance and enhancing the landscape of targeted therapy for HCC.

### Resistance to sorafenib

Noncoding RNAs (ncRNAs) and methylation are considered to be important from an epigenetic perspective. *Xu *et al*.* discovered an increase in circRNA-SORE expression within HCC cells resistant to sorafenib, which could be overcome by silencing circRNA-SORE. They further revealed that circRNA-SORE binds to the main oncogenic protein, YBX1, ensuring its persistent presence and promoting its oncogenic role [445]. *Zhang *et al*.* observed that highly metastatic HCC cells expressed higher levels of lncRNA SNHG3 than low metastatic cells. Their results showed that SNHG3 may regulate sorafenib resistance by suppressing miR‐128's function [446]. *Lin *et al*.* found that METTL3, a primary m^6^ A methyltransferase, was considerably down-regulated in human sorafenib-resistant HCC. METTL3 targets FOXO3, whose 3'-untranslated region is stabilized by m^6^ A modification of FOXO3 mRNA. They revealed that METTL3 deletion increases sorafenib resistance by eliminating METTL3-mediated FOXO3 mRNA stabilization [447].

ATP-binding box (ABC) transporters may transport sorafenib from tumor cells through exosomes, causing resistance. ABC transporters interact with sorafenib [448]. *Giacomo *et al*.* found that blocking ABC pumps with β-caryophyllene oxide (CYRO) chemosensitized liver cancer cells to sorafenib in mice [449]. *Xu *et al*.* found that exosomes propagated circRNA-SORE-mediated resistance in HCC cells [445].

Sorafenib resistance has also been linked to regulated cell death (RCD) such as ferroptosis and autophagy, as well as tumor microenvironments such as hypoxia. *Gao *et al*.* found that sorafenib-induced ferroptosis was prevented by YAP/TAZ. They further confirmed that YAP/TAZ directly and indirectly induce the expression of *SLC7A11*, a key antagonist of ferroptosis, by maintaining the stability and the ability to transcribe of ATF4 [450]. *Lai *et al*.* linked transactivation response element RNA-binding protein 2 (TARBP2) to resistance. Autophagic-lysosomal proteolysis downregulate *TARBP2*, stabilizing the expression of the CSC marker protein Nanog, and resulting in sorafenib resistance in HCC cells [451]. Hypoxia is an important feature of solid tumors, including HCC [22]. Given that the angiogenic role of sorafenib causes hypoxia within tumor tissues, *Liang *et al*.* found that hypoxia protects HCC cells against sorafenib by increasing HIF-1α expression compared to sorafenib-sensitive or untreated HCC. HIF-1α is crucial for resistance in hypoxia-inducible microenvironments [9].

In addition, tumor metabolism contributes to sorafenib resistance. *Xu *et al*.* observed decreased ROS levels in sorafenib-resistant HCC cells because sorafenib targets electron transport chain complexes to regenerate reactive oxygen species (ROS) [452]. They further confirmed the role of the UBQLN1-PGC1β axis in mitochondrial genome transcription, whose dysregulation decreases mitochondrial biogenesis and ROS regeneration, resulting in sorafenib resistance [453]. Moreover, glucose metabolism also increases sorafenib resistance. Glycolytic inhibitors and glycolytic enzyme silencing have antitumor effects in sorafenib-resistant cells [454, 455].

Additionally, *Xia *et al*.* verified FBXW2-mediated polyubiquitylation and degradation of transforming growth factor-β-activated kinase 1 (TAK1) in sorafenib-sensitive cells. However, in sorafenib-resistant cells, MTDH degrades FBXW2 mRNA, resulting in a high level of TAK1 that mediates sorafenib-resistance [456]. *Sun *et al*.* highlighted the important role of proprotein convertase subtilisin/kexin type 9 (*PCSK9*) in sorafenib resistance. They demonstrated that *PCSK9* upregulation led to the polyubiquitylation and degradation of PTEN and activated AKT, which is necessary for sorafenib resistance. This could be reversed by silencing or depletion of *PCSK9* in vivo or in vitro [457].

### Resistance to other TKIs

Currently, lenvatinib is attracting interest as a first-line treatment for HCC. Specific mechanisms of lenvatinib resistance have been linked to sorafenib resistance. Epigenetic modifications, such as methylation, play a significant role. *Wang *et al*.* found that N6-methyladenosine methylation of *FZD10* mRNA upregulated the activation of *FZD10*, which in turn stimulated MEK/ERK signaling and reduced the effect of lenvatinib on MEK/ERK phosphorylation. This effect was mediated by c-Jun through β-catenin signaling in HCC cell lines. Lenvatinib resistance was reversed by β-catenin or c-Jun knockdown [458].

Lenvatinib resistance has also been linked to ATP-binding cassette transporters. Based on a previous study, *Hu *et al*.* demonstrated that upregulation of EGFR signaling in HCC cells resulted in the upregulation of ATP-binding cassette transporter B1 (*ABCB1*) through the EGFR-STAT3-ABCB1 axis in vitro, promoting lenvatinib resistance through exocytosis, which could be reversed by the EGFR inhibitor erlotinib [459].

The correlation between apoptosis and lenvatinib in HCC has been confirmed. LncRNA MT1JP (MT1JP), a ceRNA for miR-24-3p, inhibits HCC cell apoptosis through the anti-apoptotic protein BCL2L2. Interestingly, the lenvatinib-sensitive group had lower *MT1JP* expression and higher miR-24-3p expression compared to the resistant group, indicating the importance of RCD in lenvatinib resistance [460]. *Zhang *et al*.* identified a hypoxia-mediated PPARGC1A/BAMBI/ACSL5 axis that regulates ROS generation and ferroptosis sensitivity in HCC cells. *PPARGC1A* overexpression increased lenvatinib sensitivity in patient-derived HCC organoids, suggesting an anti-tumor role [461].

Additionally, drug resistance in HCC treatment was also found in the second-line targeted agent regorafenib. In vitro experiments indicate that Glucose-6-phosphate dehydrogenase (G6PD) in HCC cells is closely related to drug resistance. As the rate-limiting enzyme of the pentose phosphate pathway (PPP), increased G6PD can induce cell resistance to regorafenib, whereas inhibition of G6PD expression can increase cell sensitivity to regorafenib. It was also confirmed that G6PD regulates the PI3K/AKT pathway, providing a basis for targeting the feedback loop between the PPP and PI3K/AKT pathway to resolve drug resistance [462]. Additionally, *Wang *et al*.* discovered that topoisomerase IIα (*TOP2A*) in HCC cells is closely associated with resistance to regorafenib, showing increased expression upon regorafenib induction. Silencing TOP2A can reverse this resistance, indicating the potential of targeting TOP2A to enhance the effectiveness of regorafenib treatment in HCC by overcoming drug resistance [463].

Therefore, progression has been achieved to address the issue of TKI resistance. However, additional advancements are required for more progression in overcoming drug resistance.

## Conclusion

The approval of sorafenib by the FDA in 2007 marked the beginning of the era of targeted therapy for liver cancer, offering hope for improved survival in patients with advanced liver cancer and inspiring optimism for the future of targeted treatments. This article offers a comprehensive review of the signaling pathways involved in the carcinogenesis of liver cancer. These pathways can be categorized into two groups: growth factor receptor-related signaling pathways (e.g., VEGFR, FGFR, TGFβR, EGFR, IGFR, and c-MET) and growth factor receptor-independent signaling pathways (e.g., Wnt-β-catenin, JAK/STAT, Hedgehog, Hippo, Notch, and NF-κB). These signaling pathways are closely associated with cell proliferation, survival, and apoptosis inhibition. Therefore, dysregulation of them is recognized as a pro-tumor factor in liver cancer pathogenesis. Additionally, we offer a comprehensive compilation of ongoing research on targeted therapies for specific pathways, highlighting the most promising developments in the field. Despite the fact that numerous targeted therapies have entered the clinical trial stage, aiming at diverse pathways, the outcomes of many still remain ambiguous regarding the efficacy of liver cancer treatments, providing scant guidance. Hence, the innovation and enhancement of targeted medications for liver cancer demand further progress.

Meanwhile, the resistance of HCC to TKIs treatment has also underscored its severity. For example, resistance to sorafenib typically develops within six months of treatment [442], indicating that TKIs alone may be insufficient to effectively reverse tumor progression. Although we have identified the common carcinogenic pathways involved in the development of HCC, the inherent complexity of liver cancer means that these pathways demonstrate variability among patients. Consequently, individuals might exhibit varied responses to identical treatments. This can be demonstrated through some clinical trials (NCT01351103, NCT04665206 etc.), which reflect a more specific selection of participants, aiming to maximize therapeutic efficacy. Additionally, given the dual role of the TGF-β pathway, it is plausible that some pathways may have a kind of intrinsic balance which is still unclear. Our present understanding may be constrained. Besides, taken the interplay of pathways into consideration, there exists a possibility that intervening directly to inhibit a component of one pathway could disturb the existing equilibrium due to human interference, potentially resulting in unpredictable outcomes. These considerations necessitate further reflection.

To further the progress of targeted therapy in liver cancer, it is crucial to explore the fundamental processes in detail and develop new targeted agents based on a comprehensive understanding of pathways, as well as to overcome challenges such as TKI resistance. Paramount to this effort is the need to formulate personalized treatment plans based on patient conditions which are judged by certain biomarkers and strategically combine different types of drugs to maximize therapeutic efficacy, thereby genuinely improving the prognosis of liver cancer patients.

## Data Availability

All data used to support the findings of this study are included within the article.
